# Sono-enzymatically engineered tannic acid/doxycycline-lignin nanoparticles enable self-assembled bioactive chitosan hydrogels for chronic wound healing

**DOI:** 10.1016/j.mtbio.2026.103792

**Published:** 2026-10-28

**Authors:** Reza Ahmadi, Eva Ramon, Katerina Todorova, Zdravka Petrova, Dolores Hinojosa-Caballero, Tzanko Tzanov

**Affiliations:** a Group of Molecular and Industrial Biotechnology, Department of Chemical Engineering, Universitat Politècnica de Catalunya, Rambla Sant Nebridi, 22, Terrassa, 08222, Spain; b Institute of Experimental Morphology, Pathology and Anthropology with Museum, Bulgarian Academy of Sciences, Acad. G. Bonchev Str., Bl. 25, 1113, Sofia, Bulgaria; c Unitat de Ferides Complexes, Consorci Sanitari de Terrassa Hospital de Terrassa, Ctra. Torrebonica, s/n, 08227, Terrassa, Spain

**Keywords:** Lignin nanoparticles, Self-assembled hydrogels, Matrix metalloproteinase/ myeloperoxidase inhibition, Antibiofilm activity, Chronic wound management

## Abstract

Chronic wounds remain a major clinical challenge due to their multifactorial pathology, characterised by excessive proteolytic and oxidative enzyme activity, persistent inflammation, bacterial infection, and biofilm formation. Here, we report a green, nano-enabled hydrogel fabricated through the *in situ* self-assembly of thiolated chitosan (TCS), driven by tannic acid/doxycycline-functionalised lignin nanoparticles (PLD NPs). Doxycycline was incorporated at a sub-inhibitory concentration (12.5 μg/mL), enabling PLD NPs (0.5 mg/mL) to inhibit matrix metalloproteinase (MMP) activity by approximately 50 % without inducing antibacterial effects. Dynamic interactions between the polymer network and NPs produced injectable, self-healing hydrogels capable of stimuli-responsive NPs release under chronic wound-like conditions, including variations in pH, hydrogen peroxide, and lysozyme. *Ex vivo* evaluation using human chronic wound exudates demonstrated potent inhibition of MMP and myeloperoxidase activities by 73 % and 80 %, respectively. The hydrogels further exhibited strong antimicrobial and antibiofilm activity against *Staphylococcus aureus* and *Pseudomonas aeruginosa*, achieving 4.8- and 4.3-log reductions in bacterial viability while preventing 73 % and 65 % of biofilm formation, respectively. *In vitro* studies confirmed excellent biocompatibility with human keratinocytes and fibroblasts, together with up to 82 % cellular antioxidant activity and enhanced fibroblast migration. Importantly, in an *in vivo* full-thickness *S. aureus*-infected mouse wound model, the hydrogels achieved complete wound closure within 18 days, outperforming commercial antiseptic treatments. Overall, this work demonstrates a multifunctional hydrogel platform that simultaneously modulates the chronic wound microenvironment, controls infection and biofilm formation, reduces oxidative stress, and promotes tissue regeneration, offering a promising strategy for advanced chronic wound management.

## Highlights

• Sono-enzymatic synthesis of tannic acid/doxycycline-lignin nanoparticles.
• *In situ* gelation of thiolated chitosan triggered by the nanoparticles.
• Injectable self-healing hydrogel responds to chronic wound microenvironment.
• Hydrogel suppresses proteases, oxidative enzymes, bacterial infection and biofilms.
• Outperforms commercial antiseptics in a bacteria-infected mouse wound model.

## Graphical abstract

**Figure:**
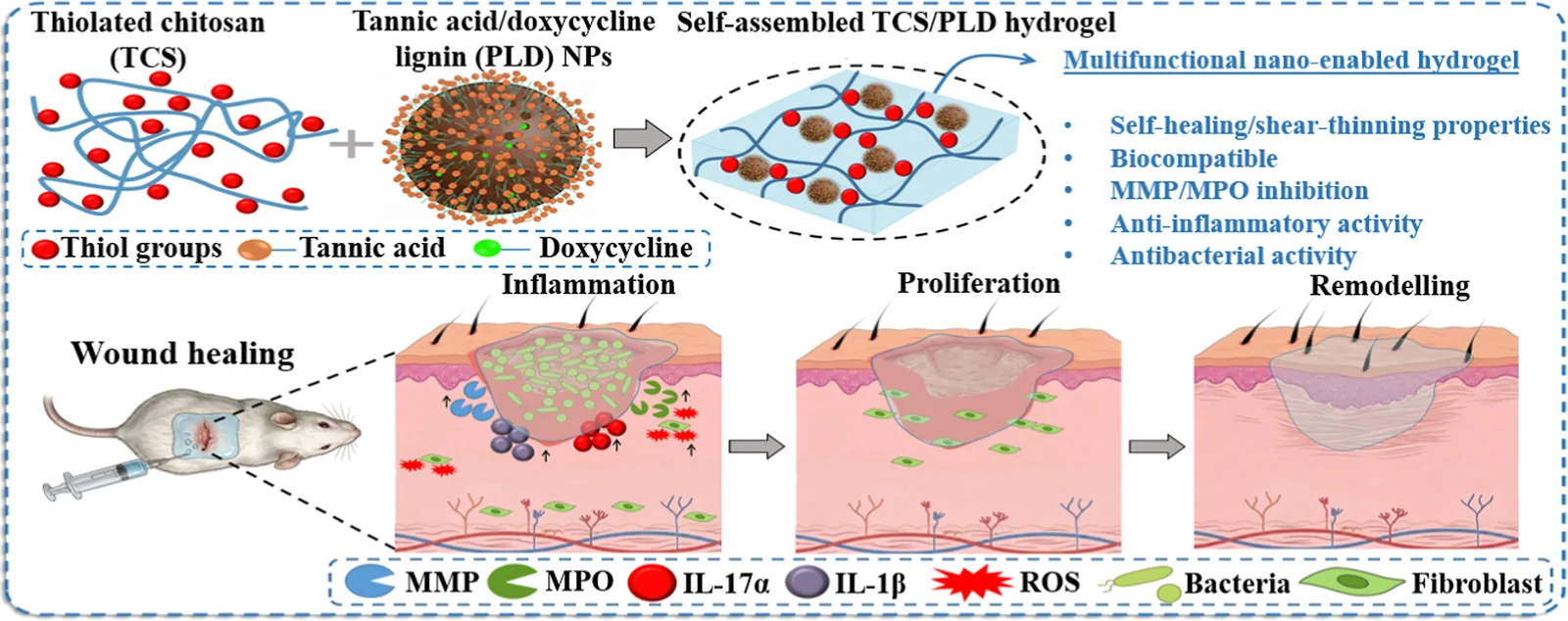

## 1 Introduction

Wound healing is a complex biological process that restores tissue integrity following injury. Disruption of the normal healing cascade during the inflammatory phase, however, can lead to chronic wounds, including venous, pressure, arterial, and diabetic ulcers [1]. These wounds impose a substantial economic burden on healthcare systems and significantly increase the risk of severe complications if left inadequately treated. In acute wounds, neutrophil-derived myeloperoxidase (MPO) produces hypochlorous acid (HOCl) for antimicrobial defence, while matrix metalloproteinases (MMPs) are tightly regulated by tissue inhibitors (TIMPs) to ensure controlled tissue remodelling, re-epithelialisation, and angiogenesis [2]. In contrast, chronic wounds are characterized by elevated MPO activity, leading to excessive production of reactive oxygen species (ROS) and HOCl. This sustained oxidative stress overactivates MMPs, disrupts the MMP/TIMP balance, and drives uncontrolled extracellular matrix (ECM) degradation [3], [4]. Moreover, chronic wounds provide a favourable environment for polymicrobial colonisation and biofilm formation [5]. These biofilms enhance antimicrobial tolerance, impair host immune responses, and delay healing by shielding microorganisms from immune clearance and limiting antibiotic penetration [6], [7].

Commercially available dressings for chronic wound management primarily absorb wound exudate and maintain a moist environment [8]. However, most fail to address the underlying pathological drivers of impaired healing, including excessive enzymatic activity, oxidative stress, bacterial infection, and biofilm formation. Although silver-based dressings have been introduced to control infection, their rapid silver release can cause cytotoxicity and exacerbate inflammation [9]. Consequently, effective chronic wound management requires multifunctional dressings that simultaneously target the diverse pathological mechanisms responsible for delayed healing [10], [11], [12] To effectively address the complexity of chronic wounds, advanced hydrogel dressings functionalised with embedded nanoparticles (NPs) have been developed [13]. For instance, a poly(vinyl alcohol) (PVA)/poly(vinylpyrrolidone) (PVP) hydrogel incorporating Ag@ZnO core–shell nanocomposites accelerated wound healing in mice by effectively inhibiting *Staphylococcus aureus* (*S. aureus*) and *Pseudomonas aeruginosa* (*P. aeruginosa*) while promoting growth factor-mediated tissue regeneration [14].

In physically crosslinked hydrogels, assembled through reversible, non-covalent interactions, NPs act as dynamic crosslinking nodes that bridge polymer chains via electrostatic interactions, hydrogen bonding, hydrophobic interactions, π–π stacking, or chain entanglement. The dynamic and reversible nature of these interactions imparts shear-thinning injectability and self-healing properties, enabling minimally invasive delivery and seamless adaptation to irregular wound geometries. Building on these advantages, our group recently developed a versatile self-assembly platform based on thiolated polymers and phenolic-shell NPs with strong interfacial activity [15]. For example, metal-phenolic network NPs composed of epigallocatechin gallate (EGCG) and cobalt have been used to drive the self-assembly of thiolated hyaluronic acid (THA) hydrogels, imparting antibacterial and antioxidant activities [16]. Guided by this rationale, our group engineered phenolic-shell lignin NPs via an ultrasound-assisted, laccase-mediated sono-enzymatic process [17] to develop phenolic-shell lignin-based NPs for self-assembled hydrogel dressings extending the self-assembled hydrogel platform to multifunctional lignin-based dressings [18].

In this study, we engineered a nano-enabled hydrogel platform for chronic wound management. Tannic acid/doxycycline-functionalised lignin NPs (PLD NPs) were synthesised through a laccase-mediated sono-enzymatic functionalisation of lignin with tannic acid (TA) and doxycycline (DOX). To selectively modulate the chronic wound microenvironment, DOX was incorporated at sub-inhibitory concentrations (<0.5 × MIC). This design choice specifically aims to leverage DOX for its potent MMP-inhibitory activity to prevent ECM degradation, while minimising its antibacterial activity and avoiding the use of DOX as an antibiotic, thereby reducing antibiotic exposure and the associated risk of antibiotic resistance [19], [20]. The resulting phenolic-shell NPs served as both structural crosslinkers and therapeutic building blocks, driving the *in situ* self-assembly of a thiolated chitosan (TCS) matrix. Importantly, the resulting hydrogels are entirely free of metals and chemical crosslinkers, providing a green and biocompatible platform for chronic wound therapy. Their rheological properties, structural stability, and stimuli-responsive release behaviour were systematically evaluated under chronic wound-mimicking conditions, including variations in pH and the presence of hydrogen peroxide and lysozyme. Therapeutic performance was further assessed *ex vivo* using human venous leg ulcer exudates by quantifying the inhibition of MMP and MPO activities. Antibacterial and antibiofilm efficacy against *S*. *aureus* and *P*. *aeruginosa* was also investigated. Finally, following confirmation of *in vitro* biocompatibility in human keratinocytes and fibroblasts, the wound-healing efficacy of the nano-enabled hydrogels was validated in an *in vivo* full-thickness *S. aureus*-infected mouse wound model.

## 2 Materials and method

### 2.1 Materials

Medical grade 15 kDa fungal chitosan (degree of deacetylation 87 %) was provided by KitoZyme (Belgium). Protobind 6000 lignin was purchased from Green Value (Switzerland). Tannic acid (TA), 3′,5′-dimethoxy-4′-hydroxyacetophenone, DOX hyclate, 2-iminothiolane hydrochloride (Traut's reagent), and the EnzChek Gelatinase/Collagenase Assay Kit were purchased from Thermo Fisher Scientific (Spain). Ellman's reagent (5,5′-dithiobis-(2-nitrobenzoic acid)), dimethyl sulfoxide (DMSO), 1,1-diphenyl-2-picrylhydrazyl (DPPH), Mueller–Hinton broth (MHB), LB agar, and Dulbecco's Modified Eagle's Medium – high glucose (DMEM) and the Proliferation Kit II XTT were supplied by Sigma-Aldrich (Spain). Neo Fetal Bovine Serum, 200 mM Glutamine and antibiotics (100U/ml Penicillin/Streptomycin) were provided by Neo Biotech, CliniScience (Spain). AlamarBlue cell viability reagent, the fluorescent probe 2′,7′-dichlorodihydrofluorescein diacetate (H_2_DCFDA), LIVE/DEAD® Viability/Cytotoxicity assay kit for mammalian cells and Live/Dead BacLight kit (Molecular Probes L7012) were obtained from Invitrogen, Life Technologies Corporation (Spain). Bacterial strains *S. aureus* (ATCC 25923) and *P. aeruginosa* (ATCC 10145), human fibroblast cells (ATCC-CRL-4001, BJ-5ta), and human keratinocyte cells (HaCaT cell line) were obtained from the American Type Culture Collection (ATCC LGC Standards, Spain). OmniPrep Solution pH 6.0, Peroxide Block, primary rabbit anti-CD3 SP7 (ready-to-use, RTU), rabbit anti-CD25 SP176 (1:200), ZytoChem Plus (HRP) Polymer Kit, and 3,3′-diaminobenzidine were all supplied by Zytomed Systems GmbH (Germany). ELISA kits for mouse IL-1β (Abbexa Ltd., Cambridge, UK) and IL-17A (FineTest, Wuhan Fine Biotech Co., Ltd., Wuhan, China) were used for cytokine quantification.

### 2.2 Synthesis and characterisation of TCS

Chemical modification of CS with thiol groups was performed as previously reported [15]. Briefly, CS was dissolved in 1 % (v/v) acetic acid to obtain a 1 % (w/v) solution at room temperature, and the pH was adjusted to 6.5 with 0.5 M NaOH. Traut's reagent was then added at CS-to-Traut's-reagent weight ratios of 5:1, 5:2, and 5:3. The reaction mixtures were purged with nitrogen to minimise thiol oxidation and stirred for 24 h at room temperature in the dark. The resulting solutions were sequentially dialysed against 5 mM HCl containing 1 % NaCl (once), 1 mM HCl containing 1 % NaCl (twice), and finally 1 mM HCl to remove unreacted Traut's reagent. The purified TCS solutions were subsequently lyophilised and stored at 4 °C until use. The thiol content of TCS was quantified using Ellman's assay. TCS (2 mg/mL) was mixed with Ellman's reagent (0.3 mg/mL) and incubated in the dark for 2 h. The samples were centrifuged at 5000 rpm for 5 min, and the absorbance of the supernatant was measured at 412 nm using an Infinite M200 microplate reader (Tecan, Austria). Thiol concentrations were determined from an L-cysteine calibration curve. Results are expressed as mean ± standard deviation (n = 3). Raman spectra were recorded using an InVia Qontor dispersive Raman microscope (Renishaw GmbH, Germany) equipped with a 532 nm laser operating at a nominal output power of 100 mW.

### 2.3 Determination of sub-minimum inhibitory concentration (MIC) of DOX

In this study, DOX was incorporated into the NPs at a sub-inhibitory concentration. To determine this concentration, *S. aureus* and *P. aeruginosa* suspensions (approximately 10^5^–10^6^ colony-forming units (CFU)/mL) in nutrient broth (NB) were incubated with DOX at concentrations ranging from 0.1 to 50 μg/mL for 24 h at 37 °C under shaking. Bacterial growth was evaluated by measuring the optical density at 600 nm, and the minimum inhibitory concentration (MIC) was defined as the lowest DOX concentration that completely inhibited bacterial growth. The sub-inhibitory concentration used for NPs preparation was subsequently established as 0.5 × MIC.

### 2.4 Synthesis of PLD NPs

First, acetosyringone (1.5 mg/mL) was dissolved in 50 mM sodium acetate buffer (pH 5) at 50 °C, followed by the dispersion of lignin (10 mg/mL). Laccase (6 U/mL) was then added, and the suspension was stirred at 50 °C for 1 h to activate the lignin. Subsequently, TA (10 mg/mL) and DOX hyclate (0.04 mg/mL) were added, and the reaction was performed under ultrasonic irradiation using a VCX 750 ultrasonic processor (Sonics) at 50 % amplitude for 1 h while maintaining the temperature at 50 °C with a circulating water bath. The resulting suspension was centrifuged three times at 18,000 × g for 20 min to remove unreacted reagents in the supernatant. The NPs pellet was resuspended in Milli-Q water and dispersed by low-intensity ultrasound. A final centrifugation at 500 × g for 5 min was performed to remove aggregated particles; the supernatant containing the PLD NPs was collected, while the pellet was discarded. The PLD NPs were stored at 4 °C until further use.

### 2.5 Characterisation of PLD NPs

The DOX content of PLD NPs was determined by quantifying the unreacted DOX remaining in the supernatant after the first centrifugation step described in Section 2.4. The supernatant absorbance was measured at 350 nm using an Infinite M200 microplate reader (Tecan, Austria). Additionally, 200 μL of PLD NPs dispersed in MilliQ water (0.125 mg/mL) was transferred to a 96-well microplate, and the absorbance at 350 nm was recorded. A calibration curve prepared using DOX hyclate was used to calculate the DOX content, which was expressed as mg of DOX per g of NPs (mg/g). The total phenolic content of PLD NPs was determined using the Folin–Ciocalteu assay as previously described [21]. UV–Vis spectra were recorded over the wavelength range of 230–500 nm to identify the characteristic absorption band of DOX grafted onto the PLD NPs. The hydrodynamic diameter, polydispersity index (PDI), and ζ-potential of the PLD NPs were measured using a Zetasizer Nano Z (Malvern Instruments Inc., UK). Particle size and morphology were further examined by field-emission scanning electron microscopy (FESEM; JEOL JSM-7001F, Japan) operating at 5 kV. The chemical structure of the NPs was characterised by Fourier-transform infrared (FTIR) spectroscopy over the range of 600–4000 cm^−1^. The surface chemical composition and elemental states of the PLD NPs were analysed by X-ray photoelectron spectroscopy (XPS) using a SPECS XPS system equipped with an XR-50 X-ray source with a twin anode (Al Kα, 1486.6 eV and Mg Kα, 1253.6 eV) and a PHOIBOS 150 EP hemispherical energy analyser coupled to an MCD-9 detector. The system included a load-lock chamber with eight-sample capacity, a sputtering unit for surface cleaning and depth profiling, and a high-pressure/high-temperature chamber for gas treatment prior to analysis. Data acquisition and spectral processing were performed using SpecsLab Prodigy software package. The binding energy scale was calibrated using the C 1s peak of adventitious carbon at 285.0 eV, and the surface elemental composition was quantified using the manufacturer's sensitivity factors.

### 2.6 Bioactivity evaluation of PLD NPs

#### 2.6.1 MMP inhibitory activity

For the MMP inhibition assay, collagenase extract (0.5 U/mL, 400 μL) was incubated with PLD NPs (50 μL) at final concentrations of 0.1, 0.5, 1.1, and 2.2 mg/mL, corresponding to DOX contents of 0.5, 2.5, 5, and 10 μg/mL, respectively, for 24 h at 37 °C. PLig NPs (without DOX) were included as controls to determine the specific contribution of DOX to the MMP-inhibitory activity of the PLD NPs. After incubation, the samples were centrifuged, and 100 μL of the supernatant was mixed with 80 μL of the kit reaction buffer and 20 μL of gelatin substrate solution (10 μg/mL). Following incubation for 10 min, fluorescence was measured at excitation/emission wavelengths of 493/528 nm. Fluorescence values from buffer-only controls (without enzyme) were subtracted from all measurements, and the sample containing collagenase alone was defined as 100 % enzyme activity.

#### 2.6.2 Zinc chelation activity

The zinc-chelation capacity of the NPs was evaluated using a zinc quantification kit to assess their potential to modulate zinc-dependent MMP activity in chronic wound conditions. Briefly, 400 μL of 1 mM Zn(NO_3_)_2_ prepared in 5 mM HEPES buffer (pH 7.0) were incubated with PLig and PLD NPs at final concentrations of 0.1, 0.5, 1.1, and 2.2 mg/mL for 12 h at 37 °C under continuous shaking (230 rpm). After incubation, the samples were centrifuged, and 50 μL of the supernatant was mixed with 50 μL of the detection reagent in a 96-well plate. The concentration of free Zn^2+^ ions was determined from a calibration curve prepared with ZnCl_2_ standards (0.1–100 μM), and fluorescence was measured at λex/em = 485/525 nm using a microplate reader.

#### 2.6.3 Antibacterial activity

The antibacterial activity of PLD NPs against *S. aureus* and *P. aeruginosa* was first evaluated by determining MIC. PLD NPs were tested over a concentration range of 0.078–2.5 mg/mL, following the protocol described in Section 2.3 The minimum bactericidal concentration (MBC) was subsequently determined by incubating bacterial suspensions in NB adjusted to an OD_600_ of 0.01 (∼10^5^–10^6^ CFU/mL) with PLD NPs for 24 h at 37 °C under shaking (230 rpm). After incubation, 10 μL aliquots from each treatment were plated onto Baird–Parker agar for *S. aureus* and cetrimide agar for *P. aeruginosa*, followed by incubation at 37 °C for 24 h. The MBC was defined as the lowest PLD NPs concentration that completely prevented visible colony formation. The antibacterial kinetics were further evaluated using overnight bacterial cultures diluted in NB to an OD_600_ of 0.01 (∼10^5^–10^6^ CFU/mL). Equal volumes (250 μL) of bacterial suspension and PLD NP suspension at the respective MIC were mixed and incubated at 37 °C under shaking (230 rpm). At predetermined time points (0, 4, 8, 12, and 24 h), samples were collected, and bacterial viability was quantified as CFU/mL using the drop plate method. Briefly, serial dilutions were prepared in PBS, and 10 μL aliquots were spotted onto agar plates. After incubation at 37 °C for 24 h, viable colonies were counted. Untreated bacterial suspensions served as controls.

#### 2.6.4 ROS generation and protein leakage assays

The ability of PLD NPs to induce intracellular ROS in bacteria was evaluated using the oxidation-sensitive fluorescent probe H_2_DCFDA. Briefly, *S. aureus* and *P. aeruginosa* cultures grown in MHB to an OD_600_ of ∼0.6 were exposed to PLD NPs at their respective MICs. After incubation of 3 h at 37 °C, the bacterial suspensions were centrifuged at 4000 × g, washed with PBS, and the pellets were resuspended in 1 mL of PBS containing 1 μL of 20 mM H_2_DCFDA. Following incubation in the dark for 30 min, fluorescence intensity was measured at λ_ex/em_ = 490/520 nm and normalised to the corresponding OD_600_ values. Untreated bacterial cultures served as controls. To evaluate bacterial membrane disruption, intracellular protein leakage was quantified using the Bradford assay, following a previously described protocol [16]. Briefly, *S. aureus* and *P. aeruginosa* suspensions (OD_600_ = 0.2) were incubated with PLig or PLD NPs for 2 h. After centrifugation at 8000 ×g for 10 min, the supernatant was collected to determine the concentration of released extracellular proteins.

#### 2.6.5 Interaction with bacterial model membranes

The interaction of NPs with bacterial model membranes was investigated using Langmuir isotherms. To mimic a Gram-negative membrane under biomimetic conditions, a phospholipid mixture of phosphatidylethanolamine (PE) and phosphatidylglycerol (PG) in an 8:2 (v/v) ratio was prepared at a concentration of 0.5 mg/mL in chloroform. Monolayers were formed in a Langmuir trough equipped with two movable barriers (KSV NIMA Langmuir−Blodgett Deposition Trough, model KN2002, Finland) maintained at 23 ± 1 °C. Surface pressure (π) was measured using a Wilhelmy plate connected to the trough. Following thorough cleaning with chloroform and water, the trough was filled with either PBS or PBS containing PLD NPs as the subphase. Subsequently, 30 μL of the lipid solution was carefully spread onto the air–liquid interface. After complete evaporation of the chloroform, surface pressure-area (π−A) isotherms were recorded at a barrier compression rate of 15 cm^2^/min. Each experiment was performed in triplicate, and a representative isotherm is presented.

### 2.7 TCS/PLD nano-enabled hydrogels synthesis

The hydrogels were prepared by the *in-situ* self-assembly of TCS and PLD NPs at room temperature. A 4 % (w/v) TCS solution prepared in 1 % (v/v) acetic acid was mixed with PLD NP suspensions at concentrations of 20, 10, or 5 mg/mL using a polymer-to-NP volume ratio of 9:1 (v/v). To investigate the effect of the thiolation degree on hydrogel properties, additional formulations were prepared using TCS synthesised at different CS:Traut's reagent ratios while maintaining a constant PLD NP concentration of 10 mg/mL (Table 1).

**Table 1:** Coding for the preparation of TCS/PLD nano-enabled hydrogels.

Hydrogels	TCS (CS: Traut's ratio - μmol/g -SH)	PLD NPs (mg/mL)	DOX content in 100 μL of NPs (μg/mL)
TCS1/PLD(20)	5:1 – 110 ± 11.28	20	10 ± 1.18
TCS1/PLD(10)	5:1 – 110 ± 11.28	10	5 ± 0.71
TCS1/PLD(5)	5:1 – 110 ± 11.28	5	2.5 ± 0.35
TCS2/PLD(10)	5:2 – 189 ± 16.41	10	5 ± 0.71
TCS3/PLD(10)	5:3 – 240 ± 14.84	10	5 ± 0.71

### 2.8 Rheological and structural characterisation of TCS/PLD hydrogels

The rheological properties of the hydrogels were characterised using a modular rheometer (MCR302, Anton Paar, Austria) equipped with electrically heated plates and a 25 mm parallel-plate geometry. Gelation kinetics were monitored by recording the storage (G′) and loss (G″) moduli of the TCS–PLD NP mixture at a constant strain of 5 % for 10 min. Oscillatory strain sweep measurements were performed at a fixed frequency of 1 Hz over a strain range of 0.1–2000 % to determine the linear viscoelastic region (LVR) and evaluate structural changes beyond the LVR. Frequency sweep measurements were subsequently carried out at a constant strain of 5 % over a frequency range of 0.1–10 Hz. The self-healing properties of the hydrogels were assessed using a three-interval thixotropy test (3iTT) with alternating strains of 5 % and 2000 %. Finally, shear-thinning behaviour was evaluated by measuring viscosity as a function of shear rate (0.01–100 s^−1^). All rheological measurements were performed at 37 °C, except for the flow measurements, which were conducted at 25 °C. The microstructure and porosity of the freeze-dried hydrogels were examined by scanning electron microscopy (Quanta 650 FEG ESEM, FEI Company, USA), and pore size distribution was quantified using ImageJ software (v. 1.5).

### 2.9 Hydrogel performance under simulated wound conditions

The PLD NP release, swelling behaviour, and stability of the hydrogels were evaluated under simulated wound conditions. The NP release was monitored by measuring the fluorescence of the incubation medium after first determining the excitation and emission maxima of the PLD NPs. Briefly, 100 mg of TCS1/PLD(10) hydrogel was incubated in 0.4 mL of 0.1 M Britton–Robinson buffer at pH 4.0, 6.0, 8.0, and 9.0. At predetermined intervals over 72 h, aliquots of the incubation medium were collected, and fluorescence was measured at λ_ex/em_ = 480/610 nm. NP release was further evaluated following incubation of the hydrogels in PBS, H_2_O_2_ (150 μM), or lysozyme (50 U/mL). The swelling behaviour and stability of the TCS1/PLD(10) hydrogel were assessed in PBS, H_2_O_2_, and lysozyme solutions using a previously reported gravimetric method [22]. Finally, after 7 days of incubation under the different pH conditions and media, the mechanical stability of the hydrogels was evaluated by strain-dependent oscillatory rheological measurements. All experiments were performed at 37 °C under continuous shaking (230 rpm).

### 2.10 Zn^2+^ chelating and BSA adsorption capacity of the hydrogels

The zinc-chelating capacity of the hydrogels was evaluated to assess *in vitro* their potential to modulate zinc-dependent MMP activity under chronic wound conditions. Briefly, 30 mg of hydrogel was incubated with 400 μL of Zn(NO_3_)_2_ solution for 12 h at 37 °C under continuous shaking (230 rpm). After incubation, the hydrogels were removed, and the concentration of free Zn^2+^ ions in 50 μL of the supernatant was determined using a zinc quantification kit. The ability of the hydrogels to adsorb bovine serum albumin (BSA) from aqueous solution was also investigated, as the molecular size of BSA is comparable to that of MMPs. Briefly, 100 mg of hydrogel was incubated in 1.5 mL of 10 mM Tris–HCl buffer (pH 7.5) containing 1 mg/mL BSA at 37 °C for 48 h. Following incubation, 20 μL of the supernatant was mixed with 200 μL of Bradford reagent in a 96-well microplate, and absorbance was measured at 595 nm. BSA concentration was determined from a calibration curve prepared using standards of 0, 50, 100, 200, 300, and 400 μg/mL. To elucidate the mechanism of Zn^2+^ chelation and BSA adsorption, the pH-dependent surface charge of the hydrogels was determined. Hydrogels were equilibrated in 0.1 M Britton–Robinson buffer at pH 4, 6, 7, 8, and 9, and their ζ-potential was measured using a Zetasizer Nano Z (Malvern Instruments, UK).

### 2.11 Radical scavenging activity of the hydrogels

The antioxidant activity of the hydrogels was assessed using the DPPH radical scavenging assay. Briefly, 30 mg of each hydrogel were incubated with 300 μL of a 60 μM DPPH solution in methanol for 3 h at room temperature in the dark. Radical scavenging activity was quantified by measuring the decrease in absorbance at 517 nm. A DPPH solution without hydrogel served as the control. The antioxidant activity was expressed as the percentage of DPPH radical scavenging.

### 2.12 Antibacterial efficacy and biofilm prevention activity of hydrogels

The antimicrobial activity of the hydrogels was evaluated against the Gram-positive bacterium *S. aureus* and the Gram-negative bacterium *P. aeruginosa* using the ASTM E2149-01 standard flask shake method. Bacterial cultures grown in Mueller–Hinton broth were diluted in sterile PBS to an OD_600_ of 0.01 (approximately 10^5^–10^6^ CFU/mL). Hydrogels (50 mg) were incubated with 2 mL of the bacterial suspension at 37 °C for 24 h under continuous shaking (230 rpm). Bacterial viability was quantified by serial dilution and agar plating to determine colony-forming units (CFU). Antibacterial activity was expressed as the log_10_ reduction in viable bacterial counts (CFU/mL) relative to the untreated control.

Bacterial morphological changes induced by the PLD NPs or hydrogels were examined by field-emission scanning electron microscopy (FESEM). Briefly, bacterial suspensions were incubated with the hydrogels for 10 h and then transferred to 48-well plates containing silicon wafers. After 24 h of incubation at room temperature, the suspensions were removed, and the bacteria adhering to the wafers were fixed overnight in a solution containing 2 % paraformaldehyde and 2.5 % glutaraldehyde. The samples were subsequently dehydrated through a graded ethanol series (25 %, 50 %, 75 %, and 100 % v/v), with each step lasting 1 h, and imaged using a FESEM (JEOL JSM-7001F, Japan) operated at 5 kV.

The antibiofilm activity of the hydrogels was evaluated against *S. aureus* and *P. aeruginosa*. Bacterial suspensions (OD_600_ = 0.01, approximately 10^5^–10^6^ CFU/mL) were prepared in Tryptic Soy Broth (TSB), and 0.5 mL was added to each well of a 48-well plate containing 100 mg of hydrogel. The plates were incubated statically at 37 °C for 24 h to allow biofilm formation. Biofilm biomass was quantified using the crystal violet assay. Following incubation, the wells were gently washed with PBS to remove non-adherent cells, dried at 37 °C for 1 h, and stained with 0.1 % crystal violet for 15 min. Excess dye was removed by washing twice with PBS, and the plates were dried for an additional 1 h. The bound crystal violet was then solubilised with 30 % acetic acid, and absorbance was measured at 595 nm. Biofilm viability was visualised using the LIVE/DEAD BacLight™ bacterial viability kit. After washing with PBS, the biofilms were stained with 100 μL of the LIVE/DEAD reagent prepared in PBS and imaged using a fluorescence microscope (Nikon Eclipse Ti-S, Netherlands). The metabolic activity of biofilm-associated bacteria was further assessed using the Cell Proliferation Kit II (XTT) according to the manufacturer's instructions. After the washing step, 0.5 mL of freshly prepared XTT/phenazine methosulfate (PMS) solution was added to each well and incubated at 37 °C for 2 h to allow metabolic reduction of the XTT reagent. Absorbance was subsequently measured at 492 and 690 nm to quantify the metabolic activity of the biofilm.

### 2.13 Biocompatibility assessment

The *in vitro* biocompatibility of the hydrogels was evaluated using human fibroblasts (BJ5ta cell line) and keratinocytes (HaCaT cell line). Cells were cultured in DMEM supplemented with 1 % (v/v) L-glutamine, 1 % (v/v) penicillin/streptomycin, and 10 % (v/v) foetal bovine serum at 37 °C in a humidified atmosphere containing 5 % CO_2_. The cells were seeded into 96-well plates at a density of 6 × 10^4^ cells per well, after which the hydrogels were placed in direct contact with the cell monolayers. Following 7 days of incubation, the hydrogels and culture medium were removed, and cell viability was assessed using the AlamarBlue assay. Briefly, cells were incubated with 150 μL of AlamarBlue reagent (10 % v/v in culture medium) for 4 h, and fluorescence was measured at at λ_ex/em_ = 550/590 nm. Cells cultured in the absence of hydrogels served as the growth control. Cell viability was further examined using the LIVE/DEAD® Viability/Cytotoxicity Assay Kit for mammalian cells. After incubation, the cells were stained with a calcein/ethidium homodimer-1 solution (3:1, v/v) prepared in PBS for 15 min in the dark. Excess stain was removed by washing with PBS, and the cells were imaged using a fluorescence microscope at λ_ex/em_ = 494/517 nm for calcein (live cells) and 517/617 nm for ethidium homodimer-1 (dead cells).

### 2.14 Cellular antioxidant activity (CAA) assay

To evaluate the ability of the hydrogels to mitigate cellular oxidative stress, the cellular antioxidant activity (CAA) assay was performed using the oxidation-sensitive fluorescent probe H_2_DCFDA. Briefly, fibroblasts were seeded in black 96-well microplates at a density of 2 × 10^4^ cells per well in 100 μL of growth medium and cultured for 24 h. After removal of the medium and washing with PBS, the cells were incubated with 200 μL of Hanks’ Balanced Salt Solution (HBSS) containing 20 μM H_2_DCFDA and 30 mg of hydrogel for 1.5 h at 37 °C. The cells were then washed with PBS, and oxidative stress was induced by adding 100 μL of 600 μM 2,2′-azobis(2-methylpropionamidine) dihydrochloride (AAPH) prepared in HBSS. Fluorescence was recorded every 5 min for 1 h at 37 °C using a microplate reader at excitation/emission wavelengths of 485/538 nm. Ascorbic acid (0.1 mg/mL in HBSS) served as the positive control. After blank subtraction and normalisation to the initial fluorescence values, the area under the fluorescence–time curve (AUC) was calculated, and the CAA was expressed as follows:

CAA unit = 100 × [1 − (∫SA/∫CA)], where ∫SA and ∫CA represent the integrated areas under the sample and control (cells treated with AAPH only) curves, respectively.

### 2.15 Cell migration assay

Scratch assays were performed to evaluate the effect of the hydrogels on fibroblast migration. Fibroblasts were seeded in 12-well plates at a density of 1 × 10^6^ cells per well and cultured for 24 h to form a confluent monolayer. A linear scratch was then created using a sterile 200 μL pipette tip, and detached cells were removed by gentle washing with PBS. The medium was replaced with 2 mL of fresh growth medium containing 100 mg of hydrogel, and the cells were incubated for 24 or 48 h to allow migration into the wound area. Following incubation, the cells were stained using the LIVE/DEAD® Viability/Cytotoxicity Assay Kit, and fluorescence images of the scratched region were acquired. Cell migration was quantified using ImageJ software by measuring the remaining scratch area and expressing it as a percentage of the initial wound area.

### 2.16 *Ex vivo* studies of hydrogel inhibitory activity on deleterious wound enzymes

The enzyme inhibitory activity of the hydrogels was evaluated *ex vivo* using wound exudate collected from dressings of patients treated at the Unit of Complex Wounds, Hospital de Terrassa. Briefly, 1 g of dressing material containing wound exudate was immersed in 5 mL of MilliQ water for 20 min and vortexed for 5 min. The resulting suspension was centrifuged three times at 18,000g for 20 min to separate the liquid exudate from the solid residue. The recovered exudate was collected and stored at 4 °C until use. MPO inhibition was determined using guaiacol as the substrate. Hydrogels (30 mg) were incubated in 200 μL of 0.1 M phosphate buffer (pH 7.5) containing 48 μL of guaiacol (167 mM) and 32 μL of 5-fold diluted wound exudate. After incubation for 1 h, the hydrogel was removed, and 150 μL of the reaction mixture was transferred to a microplate and mixed with 10 μL of 1 mM H_2_O_2_ to initiate the enzymatic reaction. Absorbance at 476 nm was recorded every minute, and MPO activity was calculated from the rate of absorbance increase. Enzyme inhibition was expressed as the percentage reduction in activity relative to the control (wound exudate and substrate without hydrogel). The inhibitory effect of the hydrogels on MMPs was evaluated by incubating 400 μL of chronic wound exudate, diluted 8-fold with PBS (pH 7.4), with the hydrogels for 24 h at 37 °C. Following incubation, 100 μL of the supernatant were mixed with 80 μL of assay reaction buffer and 20 μL of gelatin substrate solution (10 μg/mL) from the EnzChek Gelatinase/Collagenase Assay Kit. After 10 min, fluorescence was measured at λ_ex/em_ = 493/528 nm. Wound exudate without hydrogel and PBS alone served as the positive and negative controls, respectively.

### 2.17 *In vivo* wound healing with TCS/PLD hydrogel

This study was reviewed, approved, and conducted in accordance with national Regulation No. 20/01.11.2012 on laboratory animals and animal welfare and European Directive 2010/63/EU of the European Parliament. The experimental protocol was approved by the institutional ethics committee (approval No. 282, 24 September 2020). A total of fifteen 3-month-old female ICR mice were obtained from ERBOJ, Institute of Neurobiology (INB), Bulgarian Academy of Sciences (BAS). The animals were housed in the vivarium of the Institute of Experimental Morphology, Pathology and Anthropology with Museum (IEMPAM), BAS, under standard quarantine conditions. They were maintained in spacious metal cages under species-appropriate temperature and light conditions, with food and water provided *ad libitum*.

The bacterial inoculum was prepared from an *S. aureus* culture stored at −20 °C in PBS (pH approximately 7.4) supplemented with 10 % DMSO and 5 % BSA. Before use, aliquots of the bacterial suspension were thawed, thoroughly vortexed, and centrifuged twice, with an intermediate washing step in PBS.

The mice were randomly assigned to three experimental groups (n = 5 per group). All animals were sedated with xylazine, and the dorsal region from the withers to the mid-back was shaved and disinfected with 70 % ethanol. Superficial skin wounds approximately 2–3 mm in diameter were created near a skin fold using sterile surgical scissors and tweezers. Each wound was then topically inoculated with *S. aureus* at a concentration of 2 × 10^6^ CFU/μL. Topical treatment was initiated 24 h after bacterial inoculation and applied three times daily. Wounds in Group 1 were treated with TCS1/PLD(20) hydrogel, those in Group 2 with povidone–iodine solution (Braunol®, 7.5 g/100 g), and those in Group 3 with PBS. Mice in Groups 2 and 3 additionally received subcutaneous Norodrine 24 injection solution at a dose of 15 mg/kg for three consecutive days. Treatment was continued for 18 days, and the clinical condition of the animals and wound-healing progression were monitored daily until complete recovery on day 18. Four mice from each group were euthanised on day 10 for sample collection, while the remaining mouse in each group was retained to monitor complete wound recovery. Wounds were photographed at predetermined time points up to day 18 to document healing progression and generate wound-tracking profiles. Wound areas were quantified using ImageJ software, and the wound closure rate was calculated as the percentage reduction in wound area relative to the initial wound area on day 0.

Complete blood counts were performed on blood samples collected on day 10 using an automated haematology analyser (BC-2800 Vet, Mindray, China). Serum was isolated by centrifugation (1000 × *g*, 20 min) and stored at −80 °C until analysis. Serum concentrations of the pro-inflammatory cytokines interleukin-1β (IL-1β) and interleukin-17α (IL-17α) were quantified using commercially available ELISA kits according to the manufacturers' protocols. Briefly, samples and standards were added to antibody-coated 96-well microplates and incubated with biotinylated detection antibodies followed by horseradish peroxidase-conjugated reagents. After colour development with tetramethylbenzidine (TMB) substrate and reaction termination, absorbance was measured at 450 nm using a microplate reader. Cytokine concentrations were calculated from standard curves (7.81–500 pg/mL) and expressed as pg/mL.

For histopathological analysis, skin tissue samples were collected from the wound sites, fixed in 10 % neutral-buffered formalin, dehydrated through graded ethanol solutions, cleared in xylene, and embedded in paraffin. Sections (3–5 μm) were stained with haematoxylin and eosin (H&E) and examined using a Leica DM 5000B light microscope (Leica Microsystems, Wetzlar, Germany). Immunohistochemical analysis was performed on parallel 5 μm tissue sections. Following deparaffinisation and rehydration, endogenous peroxidase activity was blocked with 0.3 % H_2_O_2_ for 5 min, and antigen retrieval was carried out in citrate buffer at high temperature. The sections were incubated with rabbit primary antibodies against CD3 (SP7) and CD25 (SP176; 1:200 dilution). Immunoreactivity was detected using an HRP-conjugated polymer detection system (Agilent Technologies, Santa Clara, CA, USA), followed by visualisation with 3,3′-diaminobenzidine (DAB) and haematoxylin counterstaining. Human tonsil tissue served as the positive control, whereas negative controls were prepared by omitting the primary antibody. All sections were examined using a Leica DM 5000B light microscope.

The *in vivo* antimicrobial efficacy of the hydrogel was assessed by quantifying the bacterial bioburden in wound tissues and comparing it with that of the commercial reference treatment. On day 10, wound tissues were collected, homogenised, suspended in 10 mL of PBS, serially diluted, and plated onto blood agar supplemented with 5 % horse blood and mannitol salt agar (MSA). After incubation, colony-forming units (CFU) were enumerated. Bacterial isolates were identified by Gram staining and standard biochemical tests. Bacterial growth was classified as heavy, moderate, scanty, or absent, whereas antibiotic susceptibility was categorised as susceptible (S), susceptible with increased exposure (I), or resistant (R).

### 2.18 Statistical analysis

All data are presented as mean ± standard deviation (SD). Statistical analyses were performed using one-way analysis of variance (ANOVA) followed by Tukey's post hoc test for multiple comparisons. Differences were considered statistically significant at *P* < 0.05. Data analysis and figure preparation were carried out using OriginPro software (OriginLab Corporation, USA).

## 3 Results and discussion

### 3.1 Synthesis and characterisation of PLD NPs

DOX, a member of the tetracycline family, is well known not only for its broad spectrum antibacterial activity but also for its ability to inhibit MMPs at sub-inhibitory concentrations [23]. In this study, DOX was incorporated into the PLD NP formulation to exploit its MMP-inhibitory activity while minimising its antibacterial effect. Sub-inhibitory concentrations, defined as doses below 0.5 × MIC, do not suppress bacterial growth but can effectively modulate biological processes, such as enzymatic activity. The MIC of DOX was determined to be 25 μg/mL for *S. aureus* and 50 μg/mL for *P. aeruginosa* (Fig. 1A). Therefore, concentrations below 12.5 μg/mL (0.5 × MIC) were considered sub-inhibitory. MMP inhibition assay demonstrated a clear dose-dependent response, with DOX concentrations ranging from 0.8 to 12.5 μg/mL increasing MMP inhibition from 8.5 to 90 %, highlighting its strong inhibitory efficacy even at low, non-antibacterial concentrations (Fig. 1B).

**Fig. 1:**
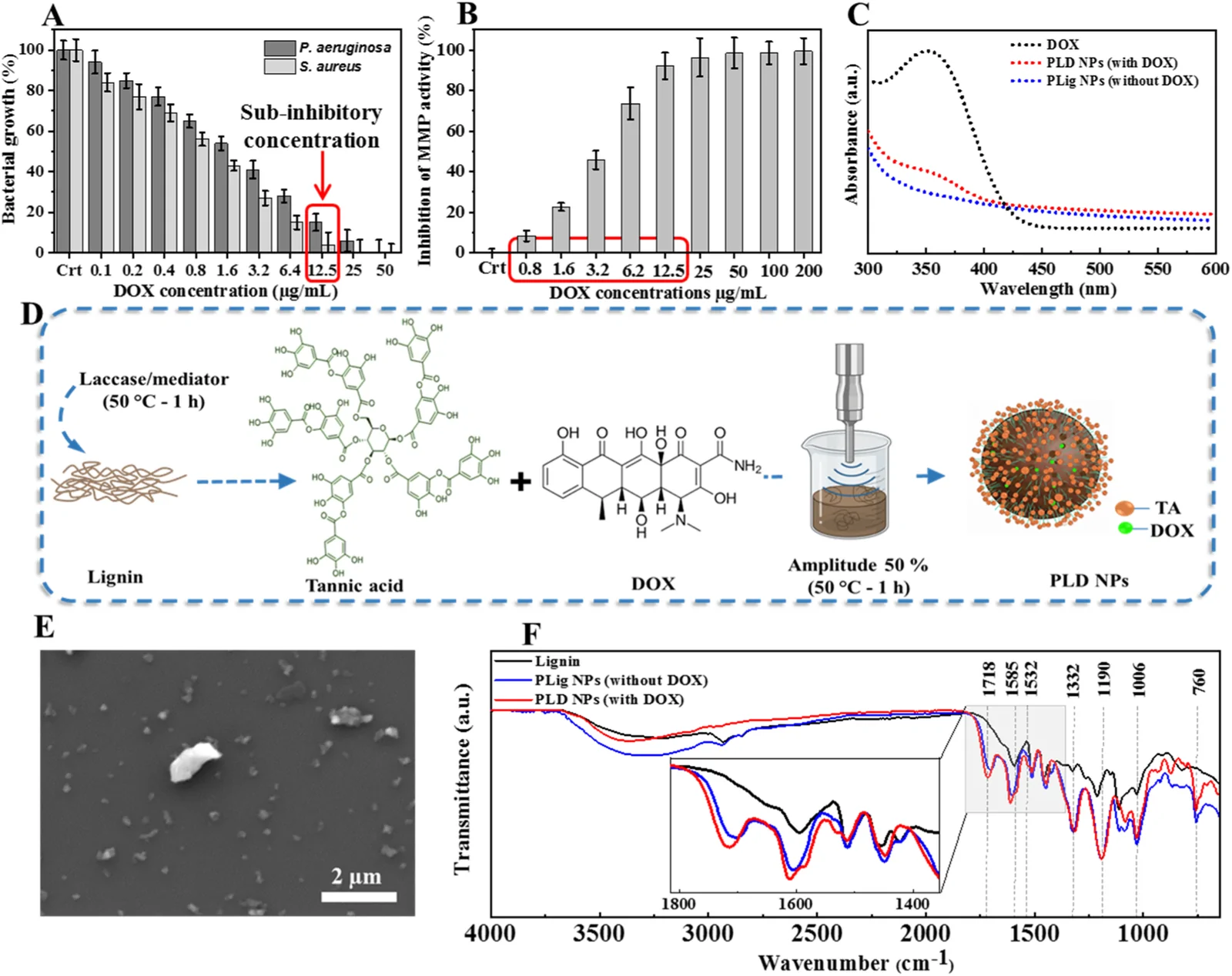
(A) MIC determination of DOX against *S. aureus* and *P. aeruginosa* to establish sub-inhibitory concentrations, (B) evaluation of MMP inhibition activity of DOX at sub-inhibitory concentrations, (C) UV–Vis spectroscopy analysis confirming DOX incorporation, (D) schematic of sono-enzymatic laccase/mediator functionalisation of bulk lignin with TA and DOX (PLD NPs), (E) FESEM micrograph of PLD NPs, and (F) FTIR spectra of lignin, PLig (without DOX), and PLD NPs.

PLD NPs were synthesised through a sono-enzymatic process, in which bulk lignin was simultaneously functionalised with TA and DOX while being converted into NPs (Fig. 1D). Lignin, TA and DOX all contain phenolic moieties that can participate in laccase-catalysed oxidative coupling reactions. Initially, laccase oxidises the phenolic groups of lignin to highly reactive phenoxy radicals, which serve as active sites for subsequent grafting reactions. Under simultaneous ultrasonic irradiation, these radicals undergo oxidative coupling with TA and DOX, resulting in the covalent functionalisation of the lignin matrix [17]. Concurrently, ultrasound-induced acoustic cavitation generates intense local shear forces and microjets that disrupt lignin aggregates, promoting their fragmentation and reassembly into stable nanoscale particles. This one-pot sono-enzymatic approach therefore enables the simultaneous nanoparticle formation and biofunctionalisation of lignin, yielding multifunctional PLD NPs with incorporated TA and DOX.

The successful functionalisation of the lignin NPs with DOX was confirmed by the appearance of a characteristic absorption shoulder at 350 nm in the UV-Vis spectrum (Fig. 1C). The DOX loading content was determined to be 0.5 mg/g of PLD NPs (0.05 % w/w), corresponding to a grafting efficiency of 28 % relative to the initial DOX input, as quantified by measuring the unreacted DOX remaining in the supernatant using a DOX hyclate calibration curve. The total phenolic content increased from 138 ± 10.16 mg GAE/g for unmodified lignin to 237 ± 13.84 mg GAE/g for PLig NPs and 245 ± 15.56 mg GAE/g for PLD NPs, with the increase primarily attributed to TA incorporation into the lignin matrix [24]. DLS analysis revealed an average hydrodynamic particle size of approximately 300 nm, while the NPs exhibited a zeta potential of −32.5 mV, indicating good colloidal stability (Fig. S1A and B). SEM further showed that the PLD NPs possessed an irregular, non-spherical morphology (Fig. 1E and S1C), consistent with the particle size determined by DLS.

FTIR spectroscopy was performed to verify the successful grafting of TA and DOX onto lignin (Fig. 1F). Compared with unmodified lignin, both PLig and PLD NPs exhibited an increased intensity of the broad O–H stretching band (3000–3600 cm^−1^), indicating an enrichment of phenolic hydroxyl groups following TA grafting. The unconjugated C=O stretching band at 1718 cm^−1^ also became more pronounced after modification, confirming the incorporation of TA into the lignin structure [25]. In the PLD NPs, this band showed a further slight increase, likely due to overlap with the amide I (C=O stretching) vibration of DOX [26]. In addition, two weak shoulders at 1585 and 1532 cm^−1^ appeared exclusively in the PLD NPs spectrum and were assigned to aromatic ring vibrations and the amide II band of DOX, respectively, providing further evidence of successful DOX functionalisation [27]. The characteristic bands at 1332 and 1190 cm^−1^, attributed to C–O stretching and phenolic O–H bending vibrations, further confirmed the presence of TA-derived phenolic functionalities in both PLig and PLD NPs [17]. Finally, the absorption bands at 1006 and 760 cm^−1^, assigned to aromatic C–H out-of-plane bending vibrations, were preserved after functionalisation, indicating that the aromatic lignin framework remained intact [28].

High-resolution XPS spectra of the C 1s, O 1s, and N 1s regions provided further evidence of the surface chemical composition of the PLD NPs (Fig. S1D). In the C 1s spectrum, the dominant peak at 284.8 eV is assigned to sp^2^-hybridised C–C/C=C bonds associated with the aromatic framework of lignin and TA. The component at 286.3 eV corresponds to C–O–C/C–OH functionalities derived from ether and hydroxyl groups, while the peak at 288.36 eV is attributed to O–C=O species originating from carbonyl or carboxyl groups [29]. In the O 1s spectrum, the signal at 533.7 eV is assigned to Ph–OH/C–O groups characteristic of lignin and TA. The peaks at 531.9 eV and 530.6 eV correspond to C=O/O=C–OH and quinone-type (Ph = O) oxygen species, respectively [30]. Importantly, a distinct, albeit low-intensity N 1s signal was detected at ∼400.1 eV corresponding to amide nitrogen [31]. As neither lignin nor TA contains nitrogen, the presence of this peak provides a strong evidence for the successful incorporation of DOX into the PLD NPs.

### 3.2 Potent MMP inhibition and antibacterial activity of PLD NPs

A key therapeutic attribute of DOX is its ability to inhibit MMPs at concentrations below its antimicrobial threshold [32]. PLig NPs (DOX-free) exhibited a concentration-dependent inhibition of MMP activity, increasing from approximately 6.5 % at 0.1 mg/mL to nearly 38.5 % at 2.2 mg/mL. This intrinsic activity is attributed to the polyphenolic functionalities of lignin and TA. Incorporation of DOX at sub-inhibitory concentrations further enhanced the MMP-inhibitory effect of the NPs. Statistical analysis showed that, across all tested concentrations, PLD NPs showed significantly greater inhibition than PLig NPs (*p* < 0.01 at lower concentrations and *p* < 0.001 at higher concentrations). At 2.2 mg/mL, PLD NPs inhibited over 90 % of MMP activity (Fig. 2A). These findings demonstrate the combined contribution of the polyphenolic lignin matrix and low-dose DOX to MMP inhibition, highlighting the strong therapeutic potential of the PLD nanoformulation for chronic wound management [33].

**Fig. 2:**
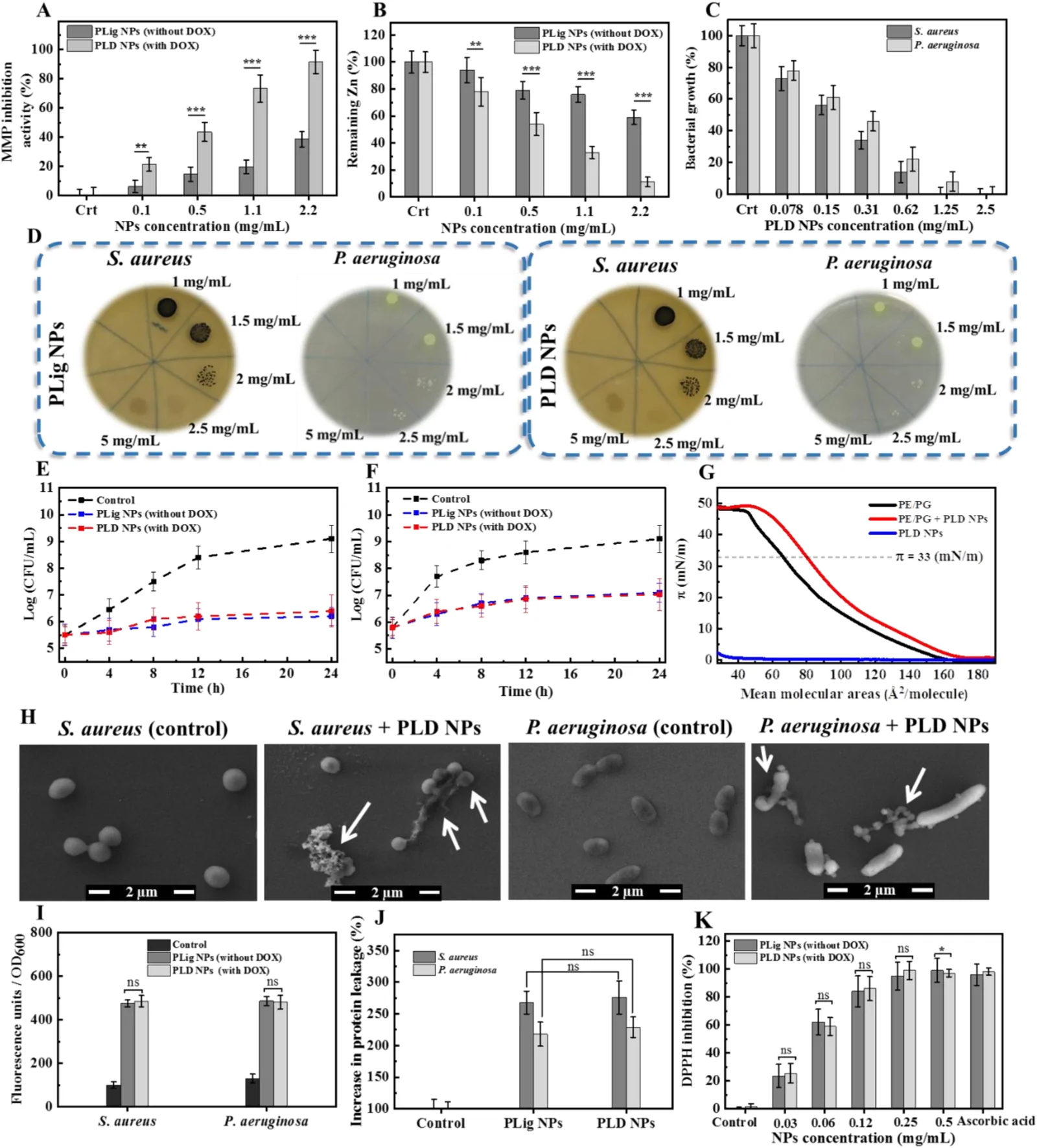
(A) MMP inhibition activity and (B) Zn^2+^ chelation assay of PLig and PLD NPs at different concentrations. (C) Determination of MIC and (D) MBC of PLig NPs (left panel) and PLD NPs (right panel) against *S. aureus* and *P. aeruginosa*. Growth kinetics of (E) *S. aureus* and (F) *P. aeruginosa* in the presence of PLig and PLD NPs at MIC concentrations (1.25 and 2.5 mg/mL). (G) Langmuir π–A isotherm analysis of a biomimetic PE/PG membrane in the presence of PLD NPs. (H) FESEM images of *S. aureus* and *P. aeruginosa* before and after exposure to PLD NPs. (I) ROS generation induced by PLig and PLA NPs at their respective MICs, and (J) protein leakage in the culture media. (K) DPPH radical-scavenging activity of PLig and PLA NPs.

MMPs are zinc-dependent endopeptidases whose catalytic activity relies on a Zn^2+^ ion located within the active site. This metal ion activates a water molecule required for the hydrolysis of peptide bonds in extracellular matrix (ECM) components [34]. Chelation of the catalytic Zn^2+^ disrupts this process, thereby reducing enzymatic activity. To investigate whether this mechanism contributes to the enhanced MMP inhibition observed for PLD NPs, their zinc-chelating capacity was evaluated. As shown in Fig. 2B, PLD NPs exhibited a concentration-dependent decrease in free Zn^2+^ and significantly greater zinc-chelating capacity than PLig NPs (*p* < 0.001). This enhanced chelation is likely due to the combined contribution of the catechol and galloyl groups present in the phenolic shell and the metal-chelating properties of DOX. These findings suggest that Zn^2+^ chelation is a major mechanism underlying the superior MMP inhibitory activity of PLD NPs.

The MIC of PLD NPs was determined to verify that the incorporated DOX made a negligible contribution to the overall antibacterial activity (Fig. 2C). The PLD NPs exhibited MIC values of 1.25 mg/mL against *S. aureus* and 2.5 mg/mL against *P. aeruginosa*, which are comparable to those previously reported for DOX-free PLig NPs [17]. These results indicate that the antibacterial activity of PLD NPs is primarily mediated by the functionalised polyphenolic shell, with only a minimal contribution from DOX [35]. The bactericidal activity of PLig and PLD NPs was further evaluated by determining the MBC against both bacterial strains (Fig. 2D). Both NP formulations exhibited comparable bactericidal profiles, with a concentration-dependent reduction in viable bacterial counts observed over the range of 1–2 mg/mL for *S. aureus* and 1–2.5 mg/mL for *P. aeruginosa*. Complete bacterial eradication was achieved at 2.5 mg/mL and 5 mg/mL, respectively, for both PLig and PLD NPs.

To further verify the negligible contribution of DOX to the antibacterial activity of PLD NPs, the growth kinetics of *S. aureus* (Fig. 2E) and *P. aeruginosa* (Fig. 2F) were monitored. For each bacterial strain, PLig and PLD NPs exhibited nearly identical growth inhibition profiles against both bacterial strains, maintaining bacterial populations at 5.5–6.5 log CFU/mL for *S. aureus* and 5.9–7.1 log CFU/mL for *P. aeruginosa*, whereas the untreated controls reached approximately 9 log CFU/mL. The close overlap of the growth curves of PLig and PLD NPs demonstrates that the antibacterial activity is primarily mediated by the functionalised polyphenolic shell, with no measurable enhancement resulting from DOX incorporation [36]. These findings confirm that DOX, at the incorporated sub-inhibitory concentration, has a negligible effect on bacterial growth. Consequently, within the PLD nanoformulation, DOX primarily functions as an MMP inhibitor rather than as an antibacterial agent.

The antibacterial mechanism of PLD NPs was investigated using complementary approaches, including NP–bacterial membrane interactions evaluated by Langmuir monolayer analysis, intracellular ROS generation, protein leakage, and FESEM imaging of bacteria following exposure to PLD NPs. The interaction of PLD NPs with a biomimetic Gram-negative bacterial membrane composed of a PE/PG monolayer was investigated using Langmuir isotherms. The interaction of PLD NPs with a biomimetic Gram-negative bacterial membrane was further investigated using Langmuir monolayers composed of phosphatidylethanolamine/phosphatidylglycerol (PE/PG). The surface pressure–area (π–A) isotherm of the control PE/PG monolayer showed the characteristic continuous increase in surface pressure until collapse at approximately 49 mN m^−1^ (Fig. 2G). As previously reported by our group, lignin-based NPs exhibit low intrinsic surface activity and do not form stable monolayers at the air–water interface, a behaviour that was also confirmed in the present study [37]. In contrast, incorporation of PLD NPs into the PE/PG monolayer shifted the π–A isotherm toward larger molecular areas compared with the control membrane. This expansion indicates that the NPs insert into and perturb the lipid packing, leading to monolayer expansion through multiple intermolecular interactions. At the physiologically relevant membrane pressure of 33 mN/m, PLD NPs increased the molecular area by approximately 11 Å^2^/molecule relative to the PE/PG monolayer alone. These changes in the π–A isotherm demonstrate that the PLD NPs disrupt the organisation of the lipid monolayer, supporting bacterial membrane perturbation as an additional mechanism underlying their antibacterial activity [38].

The ability of PLig and PLD NPs to induce intracellular ROS was assessed using the fluorescent probe H_2_DCFDA at their respective MIC concentrations, 1.25 mg/mL for *S. aureus* and 2.5 mg/mL for *P. aeruginosa* (Fig. 2I). Exposure to both NPs formulations resulted in a significant increase in intracellular ROS levels, which can promote oxidative stress, lipid peroxidation, DNA damage, and enzyme inactivation. No significant difference was observed in ROS generation between PLig and PLD NPs, indicating that this effect is solely driven by the polyphenolic shell of the NPs. These polyphenolic functionalities are known to undergo oxidation in the presence of dissolved oxygen, generating hydrogen peroxide and other reactive oxygen species that contribute to the antibacterial activity of the NPs [17]. ROS generation can contribute to oxidative damage of the bacterial membrane, potentially compromising membrane integrity and leading to the leakage of intracellular components, including proteins [16]. To further investigate this possibility, protein leakage was evaluated following exposure to PLig and PLD NPs. Both NP formulations induced a similar increase in extracellular protein levels, with protein leakage increasing by approximately 280 % in *S. aureus* and 230 % in *P. aeruginosa* compared with the untreated controls (Fig. 2J). FESEM analysis was performed to further investigate the morphological changes in bacteria following exposure to PLD NPs (Fig. 2H). Control *S. aureus* and *P. aeruginosa* cells exhibited intact and well-defined morphologies. After treatment with PLD NPs, both bacterial strains showed evident surface alterations and morphological damage, including membrane deformation and irregularities. These morphological changes are consistent with the observed protein leakage and ROS generation, supporting the involvement of membrane disruption and oxidative stress in the antibacterial activity of PLD NPs.

PLD NPs induced a concentration-dependent increase in intracellular ROS levels in both *S. aureus* and *P. aeruginosa*. At sub-MIC concentrations of 1.0 mg/mL for *S. aureus* and 2.0 mg/mL for *P. aeruginosa*, ROS generation remained low. At the respective MICs of 1.25 and 2.5 mg/mL, intracellular ROS levels increased, indicating that substantial oxidative stress was associated with antibacterial concentrations. (Fig. S2E). Moreover, DPPH assays demonstrated potent concentration-dependent radical-scavenging activity for both PLig NPs and PLD NPs, increasing from approximately 25 % at 0.03 mg/mL to approximately 98 % at 0.25 mg/mL, comparable to the activity of ascorbic acid as a positive control. No statistically significant difference was observed between the two NP formulations (Fig. 2K). The apparently opposing redox behaviours of polyphenol-rich NPs can be attributed to the context-dependent redox chemistry of their phenolic surface groups, which can act as radical scavengers or promote oxidative reactions depending on the surrounding physicochemical environment. Under the conditions of the DPPH assay, phenolic hydroxyl groups predominantly scavenge free radicals through hydrogen atom and electron transfer mechanisms, yielding marked antioxidant activity. In the presence of bacterial cells, however, the microenvironment provides redox-active biological components, dissolved oxygen, and cellular targets that favour the oxidative transformation and redox cycling of phenolic groups, thereby promoting intracellular ROS accumulation. The mildly alkaline environment characteristic of chronic infected wounds (pH 7–9) further accelerates these oxidative processes through phenolate ion formation and subsequent conversion to reactive quinone intermediates [39], [40]. Thus, the same polyphenolic surface can exhibit radical-scavenging activity under cell-free conditions while inducing oxidative stress within bacterial targets, providing a robust mechanistic basis for the dual redox behaviour of PLD NPs.

### 3.3 *In situ* self-assembly of TCS/PLD hydrogels

CS was functionalised with free thiol groups to enable hydrogel formation. Thiolation was achieved using Traut's reagent (2-iminothiolane), which selectively reacts with the primary amine groups of CS. The reaction proceeds via nucleophilic attack of the amine on the imine ring of Traut's reagent, leading to ring opening and covalent grafting of free thiol (–SH) groups onto the CS backbone (Fig. 3A). Increasing the CS-to-Traut's reagent molar ratio from 5:1 to 5:3 resulted in a higher degree of thiolation, increasing the free thiol content from 110 to 240 μmol/g (Table 1). Successful thiol functionalisation was further confirmed by Raman spectroscopy, which showed the appearance of a characteristic band at 681 cm^−1^, assigned to C–S vibrations (Fig. S2) [16].

**Fig. 3:**
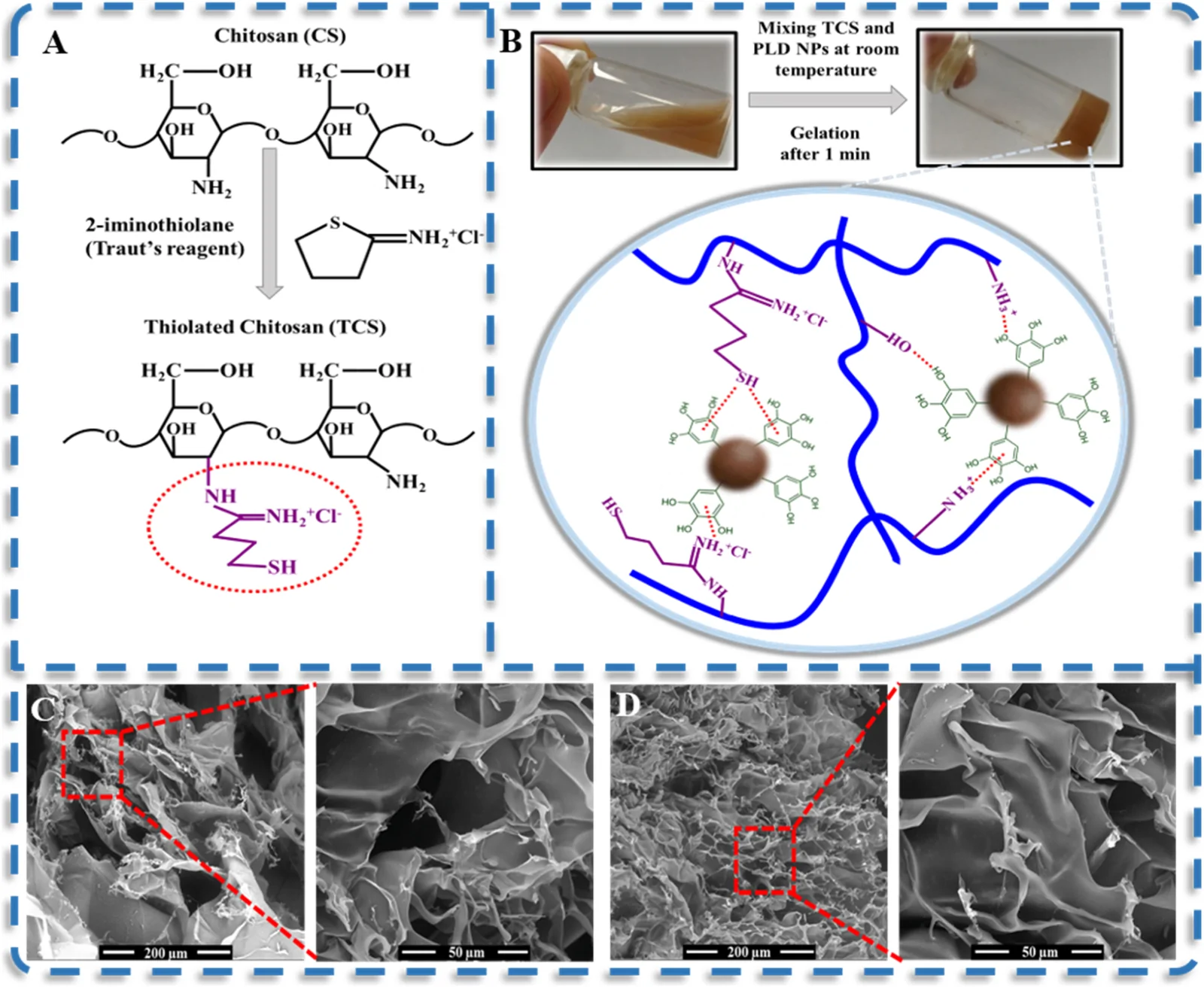
(A) Thiolation of chitosan using Traut's reagent for the preparation of thiolated chitosan (TCS). (B) Schematic of the *in situ* self-assembly of the TCS/PLD hydrogel driven by non-covalent crosslinking interactions between TCS and PLD NPs. SEM micrographs of lyophilised hydrogels with (C) high (TCS1/PLD(20)) and (D) low (TCS1/PLD(5)) PLD NPs concentration (at 500× and 1500× magnifications).

The TCS matrix was used to undergo *in situ* self-assembly with PLD NPs, which functioned both as structural crosslinking nodes and multifunctional bioactive components of the nano-enabled hydrogel. Mixing PLD NPs with the TCS solution at pH 4 and room temperature induced rapid gelation without the need for chemical crosslinkers. During self-assembly, the phenolic shell of the NPs acts as a dynamic physical crosslinker, promoting the formation of a stable three-dimensional hydrogel network. This process is driven by multiple non-covalent interactions between the catechol/galloyl-rich phenolic shell and the functional groups of TCS [15]. These include hydrogen bonding between hydroxyl groups of TCS and phenolic hydroxyl groups of the NPs, as well as interactions involving amide carbonyl groups. In addition, cation–π interactions occur between the aromatic rings of the PLD NPs and the protonated amine groups of TCS, including those introduced through 2-iminothiolane modification [41]. The network is further reinforced by thiol–π interactions between the free thiol groups of TCS and the aromatic rings of the NPs (Fig. 3B). Collectively, these dynamic non-covalent interactions enable rapid hydrogel formation while imparting the hydrogel with structural integrity, injectability, and adaptability [18]. Furthermore, oxidative coupling reactions, including Schiff base formation and Michael-type addition between oxidised phenolic groups of PLD NPs and thiol-containing moieties of TCS, may provide additional covalent stabilisation of the network [15].

The microstructure of the self-assembled hydrogels was characterised by SEM (Fig. 3C and D), revealing a porous architecture whose pore size depended on the PLD NPs concentration. Lyophilised hydrogels containing the highest NPs concentration (TCS1/PLD(20)) exhibited a mean pore size of 58 ± 22 μm, whereas those containing the lowest concentration (TCS1/PLD(5)) displayed a larger mean pore size of 110 ± 36 μm, with the difference in pore size being statistically significant (*p* < 0.05). The swelling behaviour of the hydrogels was subsequently evaluated in wound exudate over 72 h, revealing a characteristic two-stage kinetic profile, consisting of rapid water uptake during the first 24 h (Fig. 4A). All formulations exhibited a characteristic two-stage swelling profile, consisting of rapid fluid uptake during the first 24 h, followed by equilibrium. Swelling capacity decreased with increasing PLD NP concentration, from approximately 300 % for TCS1/PLD(5) to 180 % for TCS1/PLD(20). This inverse relationship is consistent with the SEM observations, indicating that higher NP contents generate a denser network with smaller pores, thereby limiting fluid uptake [42]. At a constant PLD NP concentration (10 mg/mL), TCS3/PLD(10) exhibited lower swelling than TCS1/PLD(10), suggesting that the higher thiol content promotes additional interpolymer interactions, resulting in a more tightly crosslinked hydrogel network [16]. Despite these differences, all formulations exhibited swelling capacities within the 180–300 % range, which is considered suitable for effective wound exudate management.

**Fig. 4:**
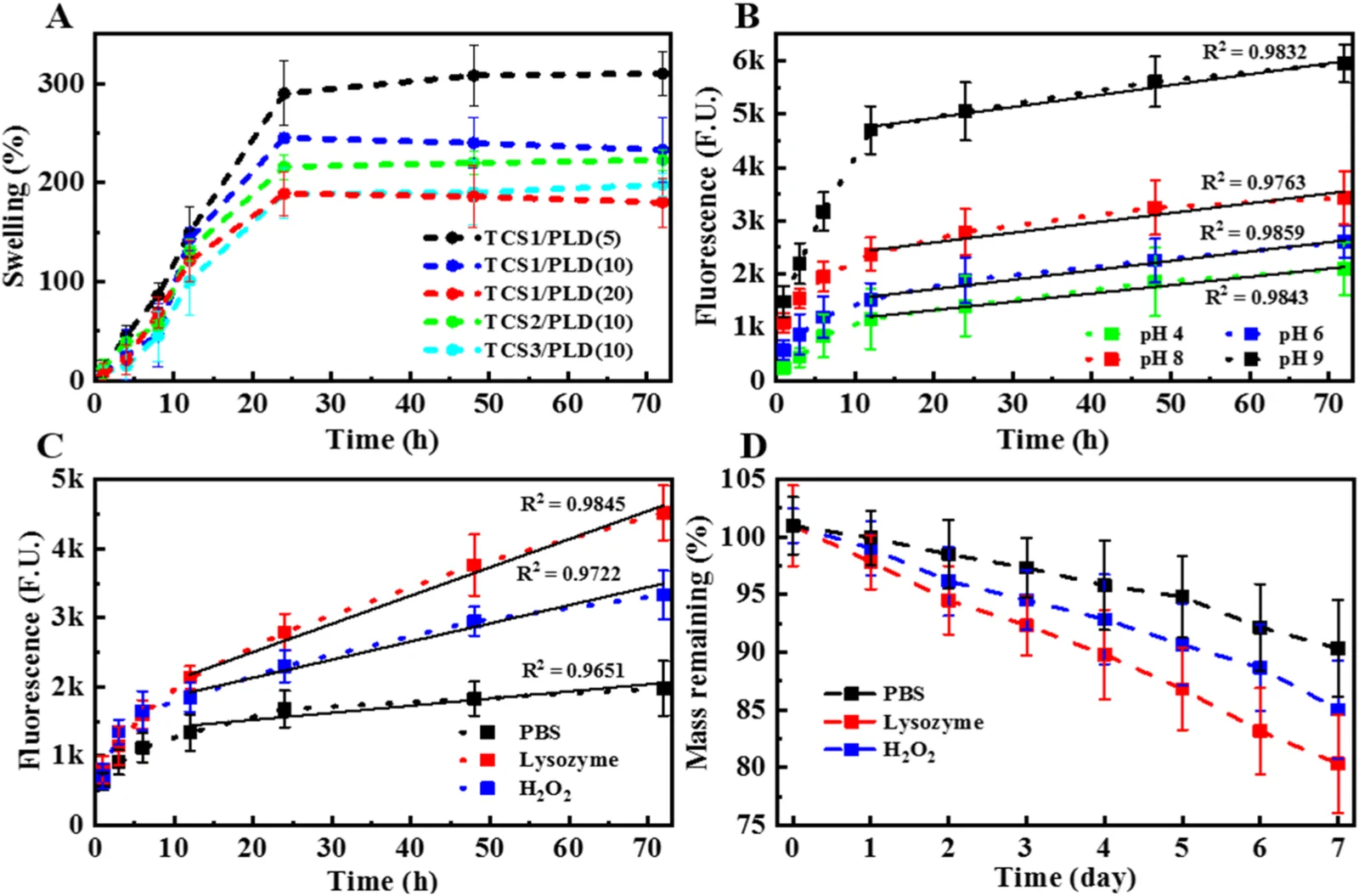
(A) Swelling capacity of the self-assembled hydrogels in wound exudate over 72 h. Stimuli-responsive PLD NPs release from the TCS1/PLD(10) hydrogel under (B) different pH conditions and (C) in PBS, H_2_O_2_-, and lysozyme-containing media over 72 h. (D) Stability assay of the TCS1/PLD(10) hydrogel in PBS, H_2_O_2_-, and lysozyme-containing media over 7 days. All tests were performed under shaking at 230 rpm and at 37 °C.

### 3.4 Stimuli-responsive release behaviour and stability of TCS/PLD hydrogels

The TCS1/PLD(10) hydrogel was selected to investigate NPs release, which exhibited a pronounced pH-dependent behaviour. Under neutral and alkaline conditions, the hydrogel displayed a biphasic release profile consisting of an initial burst release during the first 12 h, followed by a sustained release phase (Fig. 4B). Linear regression of the release kinetics from 12 to 72 h yielded R^2^ values above 0.97, indicating an approximately zero-order release profile during the sustained phase. The accelerated release at higher pH is likely attributed to the weakening of polymer–NP interactions together with the auto-oxidation of phenolic and thiol groups under alkaline conditions, which reduces the cohesion of the hydrogel network [18]. As chronic wounds are typically characterised by an elevated pH, particularly in the presence of bacterial biofilms, this pH-responsive release behaviour may enable the on-demand delivery of PLD NPs in response to the wound microenvironment, thereby enhancing therapeutic efficacy.

The NPs release profile was further evaluated in PBS and in media containing H_2_O_2_ or lysozyme, which mimic the oxidative and enzymatic conditions of infected wounds (Fig. 4C). The hydrogel exhibited both oxidative- and enzyme-responsive release behaviour, with significantly greater NP release in the presence of H_2_O_2_ and lysozyme than in PBS. The accelerated release under oxidative conditions is likely caused by the oxidation of phenolic moieties within the PLD NPs shell, which weakens the non-covalent interactions maintaining the polymer–NP network and thereby promotes NP release. Similarly, the enhanced release observed in the presence of lysozyme is attributed to the enzymatic degradation of the chitosan matrix, which facilitates the progressive disassembly of the hydrogel network. These findings demonstrate the infection-responsive behaviour of the hydrogel, whereby the elevated oxidative stress and enzymatic activity characteristic of infected wounds trigger enhanced NP release.

Hydrogels stability under chronic wound-mimicking conditions is a key factor governing the performance of wound dressings. Therefore, the stability of the TCS1/PLD(10) hydrogel was evaluated under physiological conditions by monitoring its degradation in PBS, lysozyme-containing medium, and H_2_O_2_-containing medium over 7 days at 37 °C (Fig. 4D). Hydrogel stability was quantified as the percentage of mass remaining. After 7 days, approximately 90 %, 85 %, and 80 % of the initial hydrogel mass remained in PBS, H_2_O_2_-containing medium, and lysozyme-containing medium, respectively. These findings demonstrate that the hydrogel retains substantial structural integrity under oxidative and enzymatic conditions representative of the chronic wound microenvironment, while remaining susceptible to controlled degradation that supports its stimuli-responsive behaviour.

### 3.5 Rheological characterisation of the hydrogels

The mechanical properties and gelation behaviour of the hydrogels were characterised by rheological analysis. The storage modulus (G′) and loss modulus (G″) were monitored over time under constant oscillatory conditions to evaluate the kinetics of *in situ* gelation [43]. The storage modulus (G′) reflects the elastic component of the material, whereas the loss modulus (G″) represents its viscous behaviour [44]. The transition from a liquid-like to a solid-like state is identified by the point at which G′ exceeds G″, indicating the formation of a crosslinked hydrogel network [45]. Time-sweep measurements of TCS solutions in the absence of PLD NPs showed no evidence of gelation over 10 min, with G″ remaining higher than G′ throughout the experiment. In contrast, incorporation of PLD NPs induced rapid, concentration-dependent gelation, with G′ surpassing G″ after 2.8, 1.1, and 0.4 min for TCS1/PLD(5), TCS1/PLD(10), and TCS1/PLD(20), respectively (Fig. 5A). The corresponding increase in the G′/G″ ratio also became progressively steeper with increasing NP concentration, indicating faster network formation. Increasing the degree of thiolation further accelerated gelation (Fig. 5B). At a constant PLD NP concentration (10 mg/mL), the G′–G″ crossover occurred after 0.65 min for TCS3/PLD(10), compared with 1.1 min for TCS1/PLD(10), indicating that a higher density of free thiol groups promotes more rapid interactions with the phenolic shell of the NPs. Collectively, these results demonstrate that both PLD NP concentration and chitosan thiolation are key parameters governing the kinetics of *in situ* hydrogel formation. The mechanical properties of the hydrogels were further evaluated by strain amplitude sweep measurements at 37 °C (Fig. 5C and D). All formulations exhibited typical gel-like behaviour, characterised by a broad linear viscoelastic region (LVE), indicating that their internal structure remained intact over a wide range of applied strains. Within the LVE, the storage modulus (G′) increased with increasing PLD NP concentration, demonstrating that the NPs act as effective physical crosslinking nodes and enhance the mechanical strength of the hydrogel network. Specifically, G′ increased from 11.5 Pa for TCS1/PLD(5) to 150 Pa for TCS1/PLD(20) [46]. Increasing the degree of chitosan thiolation also reinforced the hydrogel network. At a constant PLD NP concentration (10 mg/mL), G′ increased from 35.5 Pa for TCS1/PLD(10) to 65 Pa for TCS3/PLD(10), indicating that a higher density of free thiol groups promotes stronger interactions with the phenolic shell of the NPs. The transition from elastic to viscous behaviour (flow point), defined by the G′–G″ crossover, was likewise influenced by both NPs concentration and thiol content. Nevertheless, all formulations reached the flow point only at relatively high strains, demonstrating good resistance to deformation and mechanical robustness [47].

**Fig. 5:**
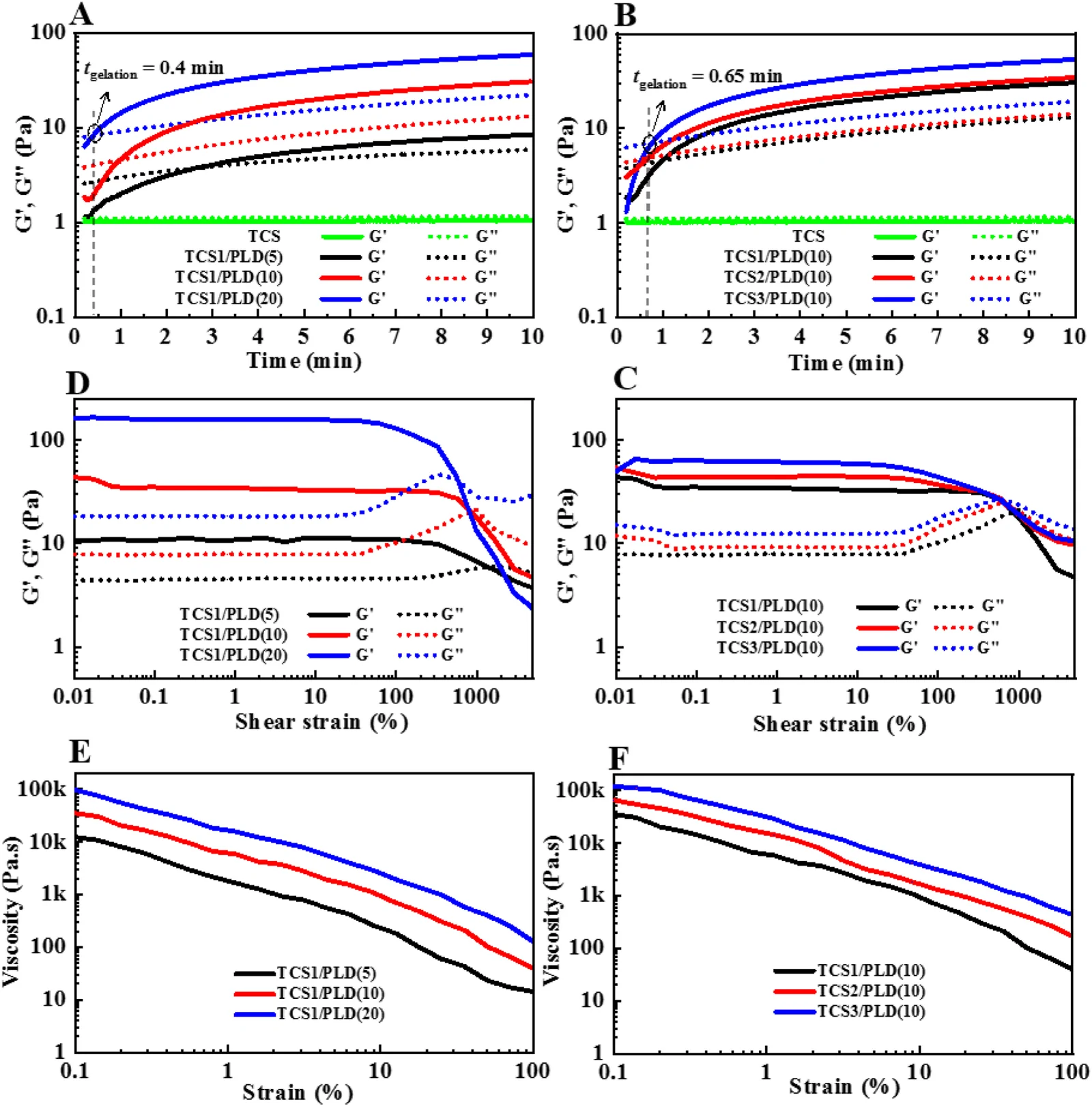
Rheological characterisation of self-assembled TCS/PLD hydrogels at 37 °C. (A and B) Time-sweep oscillatory tests monitoring gelation kinetics. (C and D) Amplitude-sweep tests defining the linear viscoelastic region (LVR). (E and F) Viscosity profiles as a function of shear rate. Panels (A, C, and E) represent hydrogel formulations with varying PLD NP concentrations, whereas panels (B, D, and F) represent formulations with different degrees of thiolation.

The injectability of the self-assembled hydrogels was assessed by measuring their viscosity as a function of shear rate (Fig. 5E and F). All formulations exhibited a marked decrease in viscosity with increasing shear rate, irrespective of PLD NP concentration or thiol content, confirming their shear-thinning behaviour. This rheological response facilitates injection through a syringe while allowing the hydrogel to readily conform to irregular wound geometries, supporting minimally invasive and precise application [48]. The self-healing properties of the hydrogels were further evaluated using a three-interval thixotropy test (3ITT) (Fig. S3A and B). Application of a high strain exceeding the flow point (G″ > G′) temporarily disrupted the hydrogel network. Upon restoring the strain to the linear viscoelastic region, both the storage modulus (G′) and loss modulus (G″) rapidly recovered to their initial values, demonstrating efficient structural recovery driven by reversible dynamic non-covalent interactions [49].

Moreover, frequency sweep analysis showed that all hydrogel formulations maintained G′ values higher than G″ over the frequency range of 0.1–100 Hz, with both moduli remaining nearly frequency-independent (Fig. S3C and D). This behaviour indicates that the nanocomposite hydrogels possess a predominantly elastic character and maintain their structural integrity under dynamic mechanical loading [16]. The rheological stability of the TCS1/PLD(10) hydrogel was further evaluated after 7 days of incubation at 37 °C under different pH conditions (pH 4, 6, 8, and 9). Strain sweep measurements confirmed that the hydrogel retained its gel-like behaviour (G′ > G″) across the entire pH range (Fig. S3E). The storage modulus (G′) increased from approximately 39 Pa at pH 4 to 55 Pa at pH 8, reaching ∼100 Pa at pH 9. This increase is likely attributed to oxidation of thiol groups and subsequent disulfide bond formation, which further reinforces the hydrogel network [18]. The hydrogel also maintained its mechanical stability after incubation in PBS, wound exudate, H_2_O_2_ -containing medium, and lysozyme-containing medium (Fig. S3F). A moderate reduction in G′ (31–33 Pa) was observed following incubation in wound exudate and lysozyme, likely due to partial degradation of the chitosan matrix. In contrast, exposure to H_2_O_2_ slightly increased G′ (approximately 47 Pa), presumably due to additional network crosslinking through oxidative thiol coupling and disulfide bond formation, as well as the oxidation of polyphenolic groups to quinones, which can react with amino groups of TCS to form Schiff base bonds. These results demonstrate that the hydrogel preserves its mechanical integrity under physiologically relevant and wound-mimicking conditions while dynamically adapting its network structure to the surrounding chemical environment.

### 3.6 Zn^2+^ chelating and BSA adsorption capacity of the hydrogels

To evaluate the Zn^2+^-chelating capacity of the hydrogels, the formulations were incubated in a Zn^2+^-containing buffer for 24 h, after which the concentration of residual free Zn^2+^ ions was quantified. Increasing the PLD NP concentration from TCS1/PLD(5) to TCS1/PLD(20) significantly reduced the amount of free Zn^2+^ remaining in solution from approximately 60 % to 12 % of the control value (100 %) (Fig. 6A). A similar trend was observed with increasing thiol content: TCS3/PLD(10), the formulation with the highest thiol content, reduced the residual free Zn^2+^ concentration to approximately 15 % of the control. These results demonstrate that both the PLD NPs and the thiolated hydrogel matrix contribute to Zn^2+^ sequestration (Fig. S4A). Chelation of Zn^2+^ disrupts the catalytic centre of MMPs, thereby inhibiting their proteolytic activity [50].

**Fig. 6:**
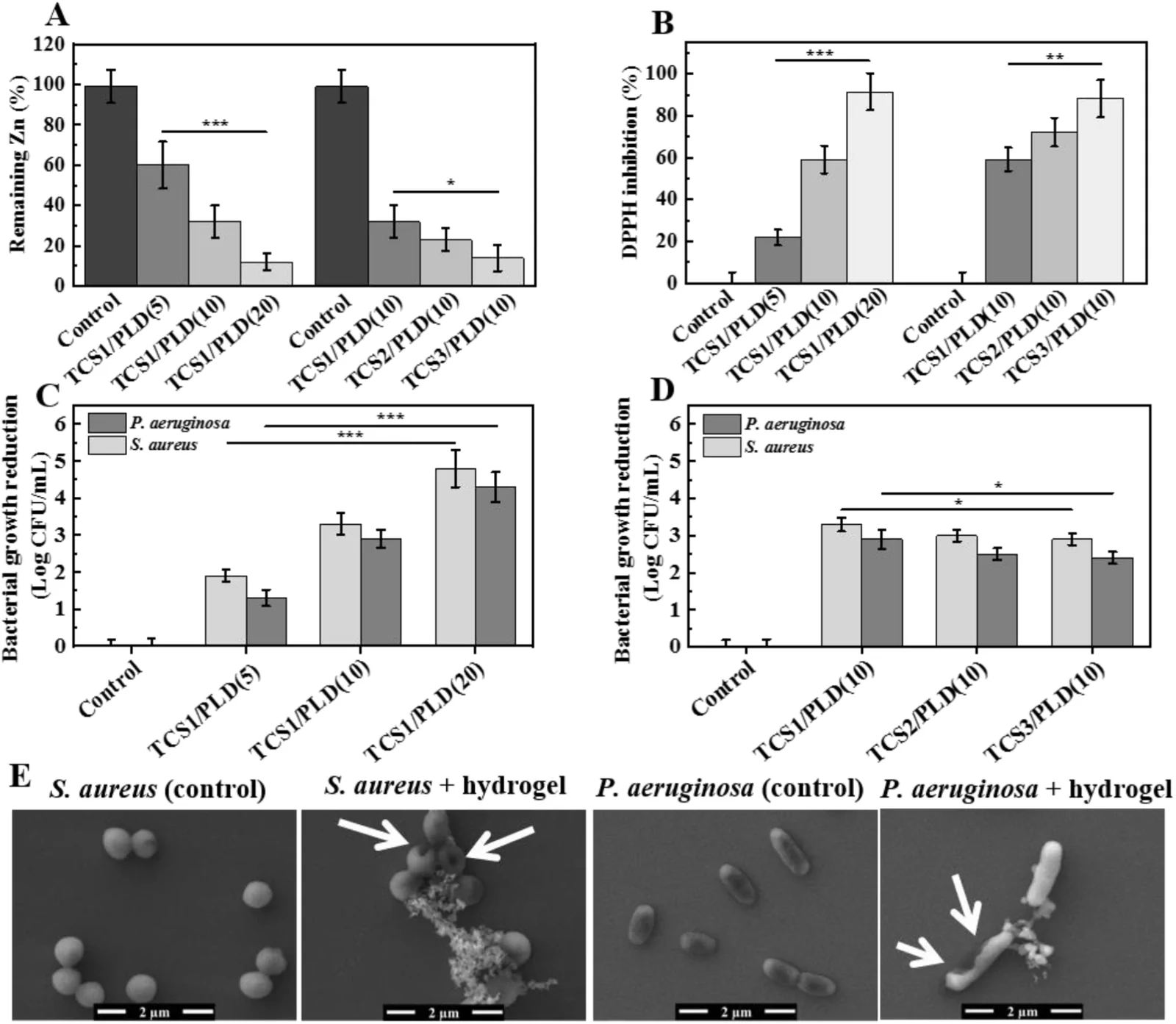
Bioactivity evaluation of TCS/PLD hydrogels. (A) Zn^2+^ chelation capacity of the hydrogels after 24 h of incubation. (B) DPPH radical scavenging activity of the hydrogels. Antimicrobial activity of hydrogels as a function of (C) PLD NP concentration and (D) thiolation degree. (E) FESEM analysis of bacterial cell membrane integrity (*S. aureus* and *P. aeruginosa*) after hydrogel treatment. All assays were performed at 37 °C.

BSA was used as a model protein to mimic the protein-rich environment of chronic wound exudates and to evaluate the protein adsorption capacity of the hydrogels. BSA was selected because its molecular weight (∼66 kDa) is comparable to that of MMPs (50–70 kDa) [51]. Protein adsorption was investigated at pH 7 over 48 h, revealing a characteristic biphasic profile similar to the swelling behaviour of the hydrogels, with rapid uptake during the first 10 h followed by a gradual approach to equilibrium (Fig. S4B). Protein adsorption decreased with increasing PLD NP concentration and degree of thiolation. TCS1/PLD(5) exhibited the highest adsorption capacity (23 mg/g), whereas TCS1/PLD(20) showed the lowest (8 mg/g). This trend is attributed to the higher crosslinking density of these formulations, which reduces hydrogel swelling and limits the free volume available for protein diffusion and entrapment. At pH 7, BSA carries a net negative charge owing to its isoelectric point (∼4.7), while the hydrogels also exhibit a negative surface charge (Fig. S4C). Consequently, electrostatic attraction is unlikely to contribute significantly to protein adsorption, indicating that protein sequestration is governed predominantly by physical entrapment within the hydrogel network [52]. To further verify protein entrapment, TCS1/PLD(10) hydrogels were incubated with BSA for 24 h, lyophilised, and analysed by FTIR (Fig. S4D). Compared with the untreated hydrogel, the spectrum showed characteristic BSA-associated features, including broadening and increased intensity of the O–H/N–H stretching band at approximately 3275 cm^−1^, enhanced C–H stretching bands between 2910 and 2870 cm^−1^, a shift and increased intensity of the amide II band (from 1510 to 1566 cm^−1^), and a more pronounced C–H bending band at approximately 1380 cm^−1^ [53]. Together, these spectral changes confirm the entrapment of BSA within the hydrogel network.

### 3.7 Radical scavenging activity of the hydrogels

To evaluate the ability of the nanocomposite hydrogels to mitigate oxidative stress, their antioxidant activity was assessed using the DPPH free radical scavenging assay (Fig. 6B). All hydrogel formulations effectively scavenged DPPH radicals, demonstrating pronounced antioxidant activity. The scavenging efficiency showed a clear dependence on PLD NP concentration, increasing from 22 % to 91 % with increasing NP content. As demonstrated in our previous studies, the polyphenol-rich shell of lignin-based NPs plays a central role in radical scavenging [15], [18]. The abundance of phenolic hydroxyl groups enables these NPs to neutralise free radicals through both hydrogen atom transfer and single-electron transfer mechanisms, generating resonance-stabilised phenoxyl radicals. The thiolated hydrogel matrix provided an additional contribution to the antioxidant activity. Increasing the thiol content enhanced DPPH scavenging from approximately 60 % for TCS1/PLD(10) to 88 % for TCS3/PLD(10), consistent with the ability of thiol groups to quench free radicals through hydrogen atom transfer. Collectively, these findings demonstrate that the antioxidant activity of the hydrogel results from the combined contributions of the polyphenol-rich PLD NPs and the thiolated chitosan matrix, providing effective protection against the oxidative stress characteristic of chronic wound environments.

### 3.8 Antibacterial activity of the hydrogels

Chronic wounds provide a favourable environment for bacterial colonisation and biofilm formation, with *P. aeruginosa* and *S. aureus* among the most prevalent Gram-negative and Gram-positive pathogens, respectively. Accordingly, the antibacterial activity of the nano-enabled hydrogel dressings was evaluated against both clinically relevant species. As demonstrated above, PLD NPs possess intrinsic antibacterial activity against both pathogens. In addition, the protonated amine groups of TCS provide a complementary antibacterial effect through electrostatic interactions with the negatively charged bacterial cell envelope. Consistent with this combined mechanism, TCS1/PLD(10) and TCS1/PLD(20) hydrogels achieved bacterial reductions exceeding 3.3 and 4.8 logs for *S. aureus* and 2.9 and 4.3 logs for *P. aeruginosa*, respectively (Fig. 6C).

Increasing the degree of thiolation resulted in a modest reduction in antibacterial activity, consistent with our previous observations [22]. This effect is likely due to the partial consumption of primary amine groups during thiolation, which decreases the density of cationic antimicrobial sites, together with the more restricted release of PLD NPs from the more densely crosslinked hydrogel network. Consequently, increasing the thiol content reduced the antibacterial efficacy of the TCS1/PLD(10) formulation from 3.3 to 2.9 logs against *S. aureus* and from 2.9 to 2.5 logs against *P. aeruginosa* (Fig. 6D).

All hydrogel formulations produced greater log reductions against *S. aureus* than against *P. aeruginosa*, consistent with previous reports demonstrating the higher susceptibility of Gram-positive bacteria to polyphenol-functionalised lignin NPs [17]. This enhanced susceptibility is also attributed to the antibacterial activity of TCS. Unlike *P. aeruginosa*, whose outer membrane constitutes an effective permeability barrier, *S. aureus* possesses a highly porous peptidoglycan cell wall that facilitates penetration of the chitosan matrix [54]. Consequently, stronger interactions between the hydrogel and the bacterial cell envelope promote greater membrane disruption, leading to leakage of intracellular components, including proteins and genomic DNA [55].

The antibacterial mechanism was further investigated by FESEM analysis of bacterial morphology following hydrogel treatment (Fig. 6E). Untreated *S. aureus* and *P. aeruginosa* cells exhibited intact membranes and well-preserved cellular morphology. In contrast, *S. aureus* cells exposed to the hydrogels displayed marked surface shrinkage and pronounced cell wall indentations, whereas treated *P. aeruginosa* cells exhibited severe membrane collapse and localised depressions while largely retaining their rod-shaped morphology. These morphological alterations are consistent with membrane damage induced by the released PLD NPs, whose polyphenol-rich shell directly interacts with the bacterial envelope [18]. The TCS matrix further contributes to this effect through its protonated amine groups, which electrostatically interact with negatively charged bacterial surfaces, increasing membrane permeability and enhancing antibacterial activity [54].

### 3.9 Biofilm prevention by the hydrogels

Biofilms are highly organised bacterial communities embedded within a self-produced extracellular polymeric substance (EPS) matrix that protects resident bacteria from antimicrobial agents and host immune responses. In chronic wounds, persistent biofilms sustain inflammation and constitute a major barrier to tissue repair [56]. Therefore, the ability of the nanocomposite hydrogels to prevent biofilm formation was evaluated under static conditions using the crystal violet assay, which quantifies total biofilm biomass. Increasing the PLD NP content significantly enhanced biofilm prevention. The TCS1/PLD(20) hydrogel reduced *S. aureus* and *P. aeruginosa* biofilm formation by approximately 73 % and 65 %, respectively (Fig. 7A). The crystal violet results were further corroborated by the XTT assay, which demonstrated a corresponding reduction in the metabolic activity of biofilm-associated bacteria. Following treatment with the TCS1/PLD(20) hydrogel, the metabolic activity of *S. aureus* and *P. aeruginosa* biofilms decreased to approximately 24 % and 33 % of the untreated control, respectively (Fig. 7C).

**Fig. 7:**
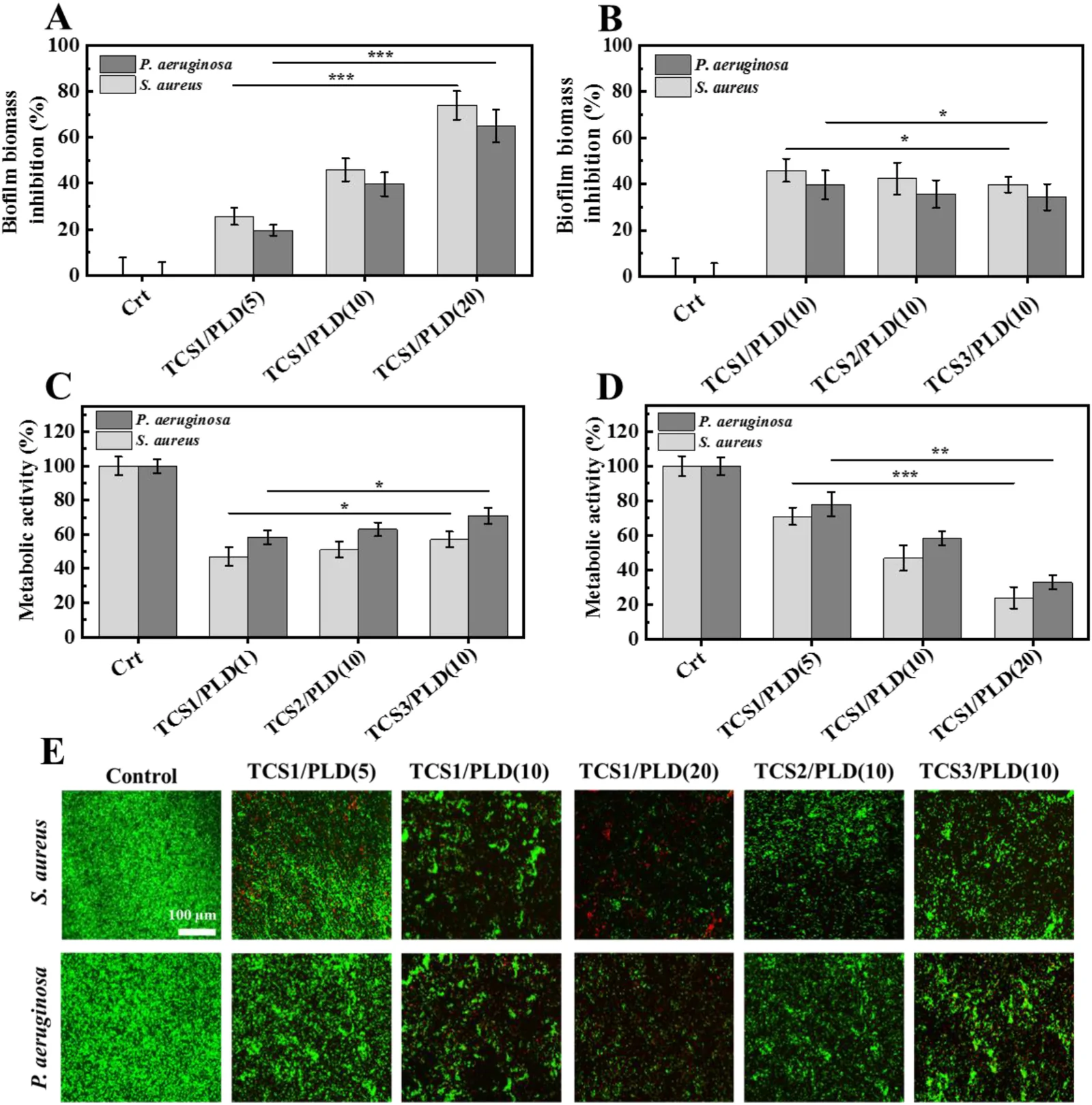
Biofilm prevention performance of TCS/PLD hydrogels. Biofilm biomass reduction of *S. aureus* and *P. aeruginosa* assessed by crystal violet assay as a function of (A) PLD NPs concentration and (B) thiolation degree. Metabolic activity of biofilm-associated bacteria assessed by XTT assay as a function of (C) PLD NPs concentration and (D) thiolation degree. (E) Live/Dead fluorescence microscopy analysis of biofilm formation, viability, and spatial distribution in the control and hydrogel-treated groups.

In contrast, increasing the degree of thiolation resulted in a modest reduction in biofilm prevention efficacy. This effect is likely associated with the reduced antibacterial activity of the hydrogels caused by two factors: (i) partial consumption of chitosan primary amine groups during thiolation, which decreases the density of cationic antimicrobial sites, and (ii) the formation of a more densely crosslinked network that restricts the release of PLD NPs. Consequently, increasing the thiol content reduced biofilm inhibition against *S. aureus* from 45 % for TCS1/PLD(10) to 39 % for TCS3/PLD(10), while inhibition of *P. aeruginosa* biofilms decreased from 39 % to 34 % (Fig. 7B). A comparable trend was observed in the XTT assay, where increasing the degree of thiolation resulted in higher metabolic activity of the remaining biofilm-associated bacteria. Specifically, the metabolic activity increased from 47 % to 56 % for *S. aureus* and from 58 % to 71 % for *P. aeruginosa*, consistent with the reduced biofilm prevention efficacy of the more highly thiolated hydrogel formulations (Fig. 7D).

Live/Dead fluorescence imaging further corroborated the quantitative biofilm assays (Fig. 7E). In the merged fluorescence images, green, red, and black regions correspond to viable bacteria, dead bacteria, and areas with minimal or no biofilm formation, respectively. Among all formulations, TCS1/PLD(20) exhibited the strongest inhibition of biofilm development, characterised by a predominance of red and black regions with only limited green fluorescence. In contrast, the untreated control displayed extensive green fluorescence, indicating high bacterial viability within a mature biofilm.

The antibiofilm activity of the hydrogels is likely mediated by multiple complementary mechanisms. The polyphenol-rich shell of the released PLD NPs may interfere with quorum-sensing (QS) signalling, disrupting bacterial communication during the early stages of biofilm development [57], [58]. In parallel, ROS generation together with increased bacterial membrane permeability promotes the elimination of planktonic bacteria before biofilm establishment through oxidative damage and leakage of intracellular components [59]. In addition, the Zn^2+^-chelating properties of the hydrogel may reduce the availability of essential metal ions required for bacterial metabolism and biofilm development. Metal ion sequestration may also compromise the structural integrity of the EPS matrix by disrupting ion-mediated crosslinking, thereby further inhibiting biofilm formation [60].

### 3.10 Biocompatibility assessment of the hydrogels

The cytocompatibility of the nanocomposite hydrogels was evaluated by the AlamarBlue assay following direct contact with human keratinocytes and fibroblasts. After 7 days of incubation, the metabolic activity of both cell types remained above 97 %, indicating negligible cytotoxicity (Fig. 8A). Cell viability and morphology were further assessed by Live/Dead fluorescence staining (Fig. 8B). The merged fluorescence images showed a high density of viable cells with normal morphology, comparable to the untreated control, as evidenced by the predominance of green fluorescence and the absence of detectable red fluorescence associated with dead cells. Together, these results demonstrate that the developed hydrogels exhibit excellent cytocompatibility and provide a favourable environment for the survival of skin cells.

**Fig. 8:**
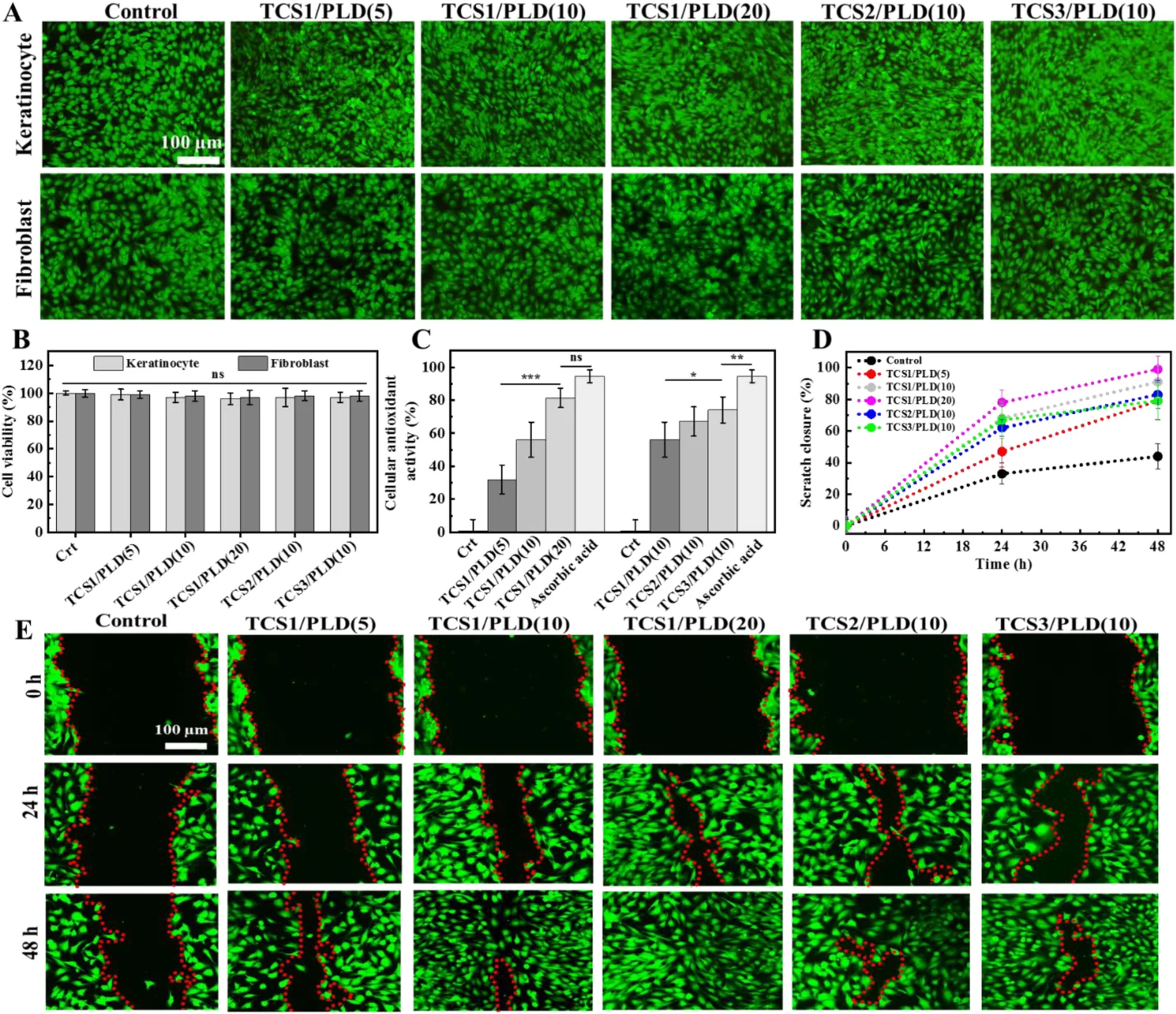
*In vitro* evaluation of TCS/PLD hydrogels. (A) Live/Dead fluorescence microscopy images of keratinocytes and fibroblasts after 7 days of incubation with the hydrogels. (B) Cell viability of keratinocytes and fibroblasts after 7 days of incubation assessed by the AlamarBlue assay. (C) Cellular antioxidant activity (CAA) of the hydrogels in fibroblasts evaluated using the DCFH-DA assay. Promoting fibroblast migration: (D) Quantitative analysis of scratch closure over 48 h and (E) Fluorescence microscopy images showing fibroblast migration into the scratch area at 0, 24, and 48 h.

### 3.11 Cellular antioxidant activity of the hydrogels

Chronic wounds are characterised by persistent inflammation and excessive production of ROS, which induce oxidative damage and impair the function of fibroblasts and keratinocytes. Accordingly, the cellular antioxidant activity (CAA) of the hydrogel formulations was evaluated in fibroblasts using the DCFH-DA fluorescent probe to determine their ability to attenuate intracellular oxidative stress. As expected, ascorbic acid, used as the positive control, exhibited the highest CAA value (94.5 %). Among the hydrogel formulations, TCS1/PLD(20) showed the greatest antioxidant activity (82 %), followed by TCS1/PLD(10) (56 %) and TCS1/PLD(5) (32 %), demonstrating a clear PLD NP concentration-dependent enhancement (Fig. 8C). Likewise, increasing the thiol content further improved the antioxidant response, with CAA values of 55 % and 73 % for TCS2/PLD(10) and TCS3/PLD(10), respectively. These findings indicate that both the polyphenol-rich PLD NPs and the thiolated chitosan matrix contribute to intracellular ROS scavenging, thereby protecting fibroblasts from oxidative stress. This cellular antioxidant activity is consistent with the results of the DPPH assay, confirming the combined antioxidant contribution of the PLD NPs and free thiol groups within the hydrogel matrix.

### 3.12 Promoting cell migration

Migration of fibroblasts, keratinocytes, and endothelial cells is essential for tissue repair and regeneration. In chronic wounds, impaired cell migration contributes to persistent inflammation and delayed wound closure. Therefore, stimulating cell migration is considered a key therapeutic strategy for promoting wound healing [61]. The ability of the nanocomposite hydrogels to promote fibroblast migration was evaluated using an *in vitro* scratch assay (Fig. 8D and E). After 24 h, all hydrogel formulations significantly enhanced scratch closure compared with the untreated control. TCS1/PLD(20) exhibited the highest closure rate (80 %), whereas TCS1/PLD(5), TCS1/PLD(10), TCS2/PLD(10), and TCS3/PLD(10) achieved wound closure rates ranging from 45 % to 70 %. After 48 h, complete closure was observed in the TCS1/PLD(20) group, while the remaining formulations reached 75–90 % closure, compared with only 45 % for the untreated control (Fig. 8D and E). Representative images of the scratch area are provided in Fig. S5. The enhanced migratory response is likely associated with the sustained release of polyphenol-functionalised PLD NPs, which have previously been shown to stimulate fibroblast activity. Polyphenolic compounds have been reported to promote cell migration through activation of intracellular signalling pathways, including protein kinase A (PKA) and p38 mitogen-activated protein kinase (p38-MAPK), which regulate cytoskeletal remodelling and cell motility [62]. These pathways have also been associated with increased expression of pro-regenerative growth factors, such as vascular endothelial growth factor (VEGF) and transforming growth factor-β1 (TGF-β1), thereby supporting tissue regeneration and extracellular matrix remodelling [63]. Together, these effects may enhance collagen deposition and accelerate wound closure.

### 3.13 *Ex vivo* inhibitory activity of the hydrogels on deleterious wound enzymes

In chronic wounds, excessive MPO activity promotes the accumulation of hypochlorous acid (HOCl), which contributes to MMP activation and ultimately drives uncontrolled degradation of the ECM [64]. Consequently, simultaneous modulation of MPO and MMP activity represents an attractive therapeutic strategy for chronic wound management. To evaluate the therapeutic potential of the hydrogels under clinically relevant conditions, wound exudates collected from chronic wound dressings, characterised by high oxidative and proteolytic activity, were used as the enzymatic source. The inhibitory performance of the hydrogels depended on both the PLD NP concentration and the thiol content of the TCS matrix. Increasing the PLD NP concentration from TCS1/PLD(5) to TCS1/PLD(20) significantly enhanced MPO inhibition from 34 % to 80 % (Fig. 9A). Likewise, at a constant NP concentration, increasing the thiol content of the matrix improved MPO inhibition from approximately 60 % for TCS1/PLD(10) to 78 % for TCS3/PLD(10) (Fig. 9B). The enhanced inhibitory activity is likely attributed to the combined contribution of released PLD NPs and the hydrogel matrix. The polyphenol-rich shell of the NPs can act both as a substrate within the MPO catalytic cycle and as a scavenger of HOCl through one-electron redox reactions, thereby attenuating MPO-mediated chlorination activity [15]. In addition, adsorption of the positively charged MPO onto the negatively charged hydrogel surface at physiological pH (Fig. S4C) is expected to promote subsequent interactions with thiol and phenolic groups, further contributing to enzyme inhibition [22].

**Fig. 9:**
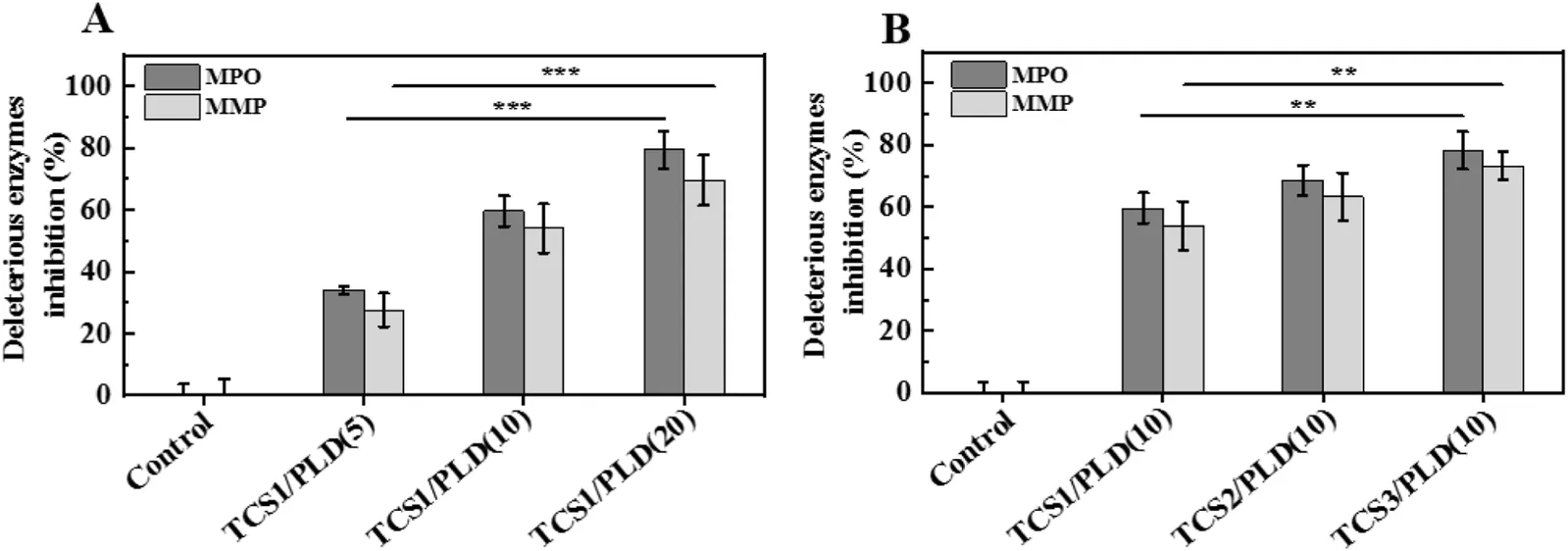
*Ex vivo* inhibition of MPO and MMP in human chronic wound exudates by the hydrogels as a function of (A) PLD NP concentration and (B) thiolation degree.

A similar trend was observed for MMP inhibition, with both PLD NP concentration and thiol content influencing the inhibitory activity of the hydrogels. Increasing the PLD NP concentration from TCS1/PLD(5) to TCS1/PLD(20) significantly enhanced MMP inhibition from 27 % to 70 % (Fig. 9A). Similarly, increasing the thiol content from TCS1/PLD(10) to TCS3/PLD(10) increased MMP inhibition from 54 % to 73 % (Fig. 9B). The observed MMP inhibition is likely the result of the combined contribution of several mechanisms. These include the presence of thiol groups within the hydrogel matrix and the release of PLD NPs carrying polyphenolic compounds and DOX, which inhibit MMP activity through Zn^2+^ chelation at the catalytic site (Fig. 2A and B). As demonstrated in the previous section, sequestration of proteolytic enzymes may represent an additional mechanism contributing to MMP inhibition. In addition, all hydrogel formulations exhibited a negative zeta potential over the pH 7–9 range (Fig. S4C), which is representative of the chronic wound microenvironment. This negative surface charge is attributed to the partial deprotonation of thiol and phenolic groups [65]. Consequently, electrostatic attraction between the negatively charged hydrogel and the positively charged MMP molecules is expected to promote enzyme adsorption, thereby increasing the local concentration of MMPs at the hydrogel surface and facilitating their inhibition.

### 3.14 *In vivo* validation of TCS1/PLD(20) hydrogel

Based on its superior *in vitro/ex vivo* performance, including potent inhibition of wound-associated enzymes, antibacterial and antibiofilm activity, high cellular antioxidant capacity, and promotion of fibroblast migration, the TCS1/PLD(20) hydrogel was selected for *in vivo* evaluation. Its therapeutic efficacy was assessed using a full-thickness *S. aureus*-infected murine wound model, with PBS serving as the negative control and Braunol as the commercial antiseptic reference treatment. Although the infected acute wound model represents a more challenging and pathologically relevant environment than an uninfected acute wound, it does not fully reproduce the complex pathology of established chronic wounds, including prolonged inflammation, underlying comorbidities, and impaired healing mechanisms. Nevertheless, it provides a biologically relevant setting in which bacterial infection may trigger pathological processes targeted by the multifunctional hydrogel developed in this study.

Gross pathological examination confirmed the successful establishment of full-thickness wounds involving both the epidermis and dermis in all experimental groups (Fig. 10A). Throughout the 18-day study, progressive wound contraction driven by the panniculus carnosus muscle resulted in a gradual reduction in wound area, with early healing characterised by fibrin clot formation. Wound-tracing analysis and quantitative wound closure measurements (Fig. 10B and C) demonstrated that the hydrogel-treated group consistently exhibited a faster rate of wound healing than both control groups. During the early inflammatory phase, no major complications were observed, although minimal purulent exudate was detected exclusively in the PBS-treated wounds. By days 9–13, complete re-epithelialisation had not yet occurred in any group; however, active granulation tissue formation was evident, with the hydrogel-treated wounds displaying a markedly smaller residual wound area than the controls. By day 18, the hydrogel-treated wounds had reached nearly complete closure and exhibited visible hair regrowth within the healed tissue, indicating advanced tissue regeneration and restoration of skin appendages. In comparison, wounds treated with Braunol and PBS achieved approximately 90 % and 80 % closure, respectively, by the end of the study.

**Fig. 10:**
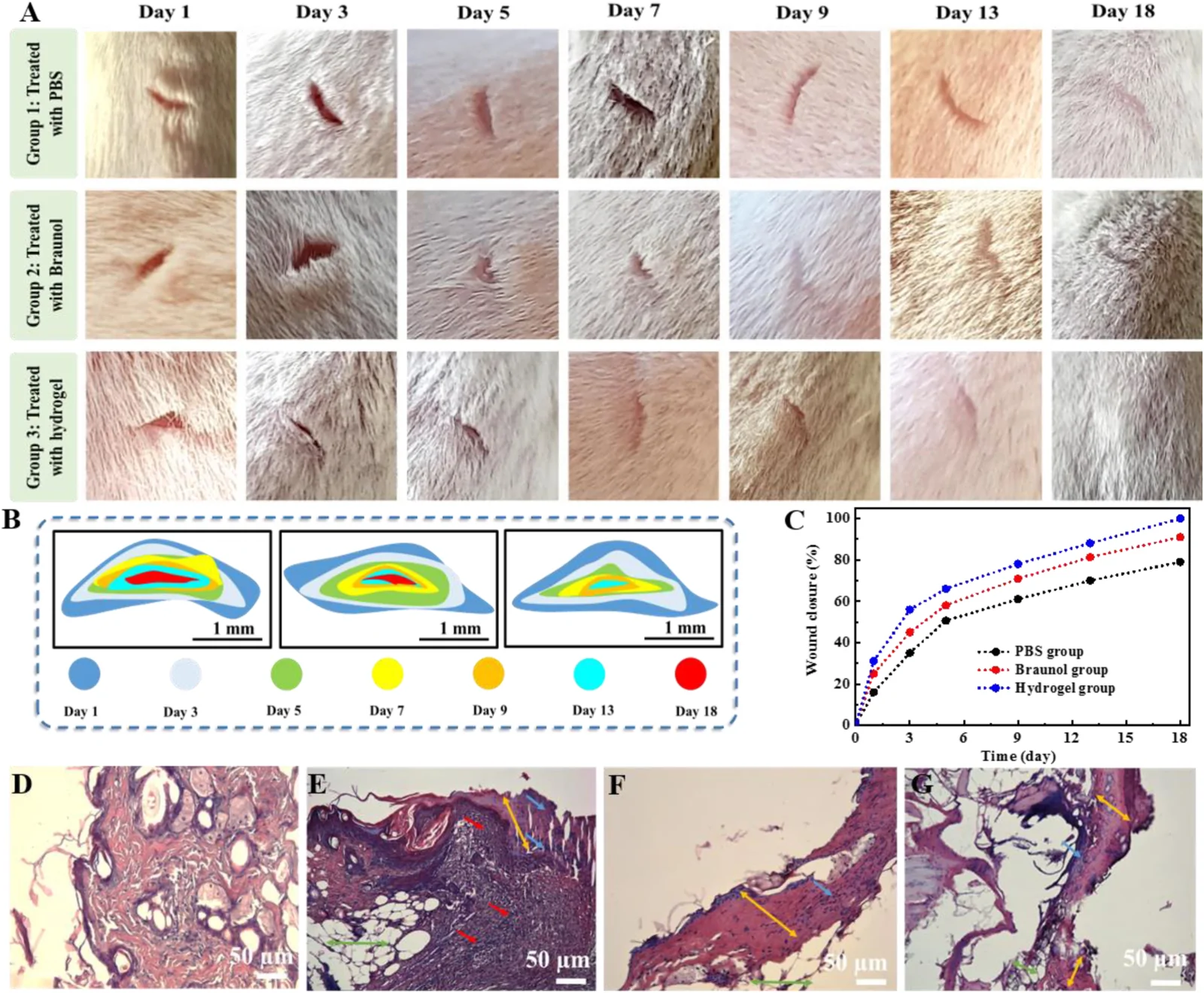
*In vivo* evaluation of TCS/PLD hydrogels in a mouse *S. aureus*-infected full-thickness wound model. (A) Photographs of wound closure in the PBS, Braunol, and hydrogel-treated groups throughout the 18-day healing period. (B) Wound-tracking traces. (C) Quantitative wound closure rates over 18 days. Histopathological evaluation of wound tissues on day 10 following H&E staining. (D) Normal skin histology. (E) PBS-treated group. (F) Braunol-treated group. (G) Hydrogel-treated group. Yellow double-headed arrows indicate granulation tissue occupying the wound bed; blue arrows indicate proliferating fibroblasts within the granulation tissue; red arrows indicate inflammatory cell infiltrates in the dermis and hypodermis; and green double-headed arrows indicate adipose tissue in the hypodermis beneath the wound area.

A subset of animals was euthanised on day 10 for histological evaluation of wound healing and collection of blood and serum samples for subsequent analyses. Histopathological examination of skin sections was performed to compare tissue regeneration in the hydrogel-treated, Braunol-treated, and PBS-treated groups (Fig. 10D–G, Table 2). The assessment focused on key indicators of wound healing, including granulation tissue formation, inflammatory cell infiltration, neovascularisation, and tissue necrosis. In all treatment groups, the wound bed contained delicate pink granulation tissue (yellow double-headed arrows), indicating active tissue repair. Abundant proliferating fibroblasts (blue arrows) and extracellular matrix deposition were observed throughout the regenerating tissue, while newly formed blood vessels were more prominent in the hydrogel and Braunol groups, suggesting enhanced neovascularisation. These two groups also exhibited only sparse inflammatory cell infiltration, within the range expected for normal wound healing, together with early signs of skin appendage regeneration. In contrast, wounds treated with PBS displayed marked inflammatory infiltrates composed predominantly of lymphocytes, neutrophils, and occasional monocytes within the dermis and hypodermis (red arrows). Adipose tissue in the hypodermis is indicated by green double-headed arrows. Importantly, histological features indicative of focal purulent inflammation following *S. aureus* infection were absent in both the hydrogel and Braunol groups on day 10. In contrast, several animals in the PBS group exhibited persistent local bacterial inflammation, characterised by lymphomonocytic and neutrophilic infiltration beneath the newly formed granulation tissue. Overall, the histopathological findings demonstrate that wound healing was more advanced in the hydrogel-treated group and was comparable to, or slightly better than, that observed following Braunol treatment.

**Table 2:** Comparative analysis of treatments based on morphological observations on day 10.

Group	*S. aureus* infection/antibiotic treatment	Inflammation	Granulation	Necrosis
Hydrogel	+/−	-	++	-
Braunol	+/+	-	++	-
PBS	+/+	+++	+	+

Understanding the physiological processes underlying normal wound repair and the pathological mechanisms responsible for delayed healing is essential for the development of effective therapeutic interventions. Physiological wound healing proceeds through four overlapping phases: haemostasis, inflammation, proliferation, and remodelling [66]. Successful healing depends on the timely resolution of inflammation and progression to the proliferative phase, whereas prolonged inflammation—often driven by bacterial infection—can impair tissue regeneration and result in chronic, non-healing wounds. Accordingly, this study focused on the inflammatory and proliferative phases of healing in *S. aureus*-infected wounds subjected to different treatments. By day 10, wounds in all groups had progressed beyond the acute inflammatory stage and exhibited granulation tissue formation, indicating entry into the proliferative phase. However, clear differences in healing progression were observed between the treatment groups. Compared with the hydrogel- and Braunol-treated wounds, the PBS group exhibited persistent infiltration of lymphocytes and neutrophils, accompanied by pronounced dermal and subdermal inflammation and focal necrotic areas. In contrast, both the hydrogel and Braunol groups displayed histological features consistent with physiological wound healing at this stage, including well-developed granulation tissue, reduced inflammatory infiltration, and early neogenesis of skin appendages. These findings are likely associated with the effective control of bacterial infection, enabling timely resolution of inflammation and progression toward tissue regeneration.

Immunohistochemical analysis further supported the histological findings, demonstrating more advanced wound healing in the hydrogel- and Braunol-treated groups than in the PBS control (Fig. 11A). As shown by the corresponding H&E sections (Fig. S6), wounds treated with PBS exhibited a dense inflammatory infiltrate, with leukocytes, including neutrophils, forming a demarcation zone beneath the granulation tissue. Neutrophils play a key role during the early inflammatory phase by phagocytosing bacteria and removing necrotic tissue, while monocytes are subsequently recruited in response to chemotactic signals and contribute to tissue repair through cytokine and chemokine secretion. The observed leukocyte diapedesis is consistent with the inflammatory response to skin injury. Activated monocytes promote the recruitment of T lymphocytes, whose infiltration typically increases from day 7 and reaches a maximum around day 14, corresponding to the transition from late inflammation to the proliferative phase [67]. T cells have also been implicated in tissue repair and dermal scar formation [68]. To assess T-cell infiltration, skin sections collected on day 10 were immunostained for CD3 and CD25 (Fig. 11A). CD3 is a pan-T-cell marker that identifies mature T lymphocytes, whereas CD25 (interleukin-2 receptor α, IL-2Rα) is expressed on activated T cells and other immune cell populations involved in regulating immune responses. Strong CD3 and CD25 immunoreactivity was predominantly observed in the PBS-treated wounds, indicating persistent immune activation. In contrast, only limited staining was detected in the hydrogel and Braunol groups, consistent with their reduced inflammatory response and with the microbiological, haematological, and histopathological findings.

**Fig. 11:**
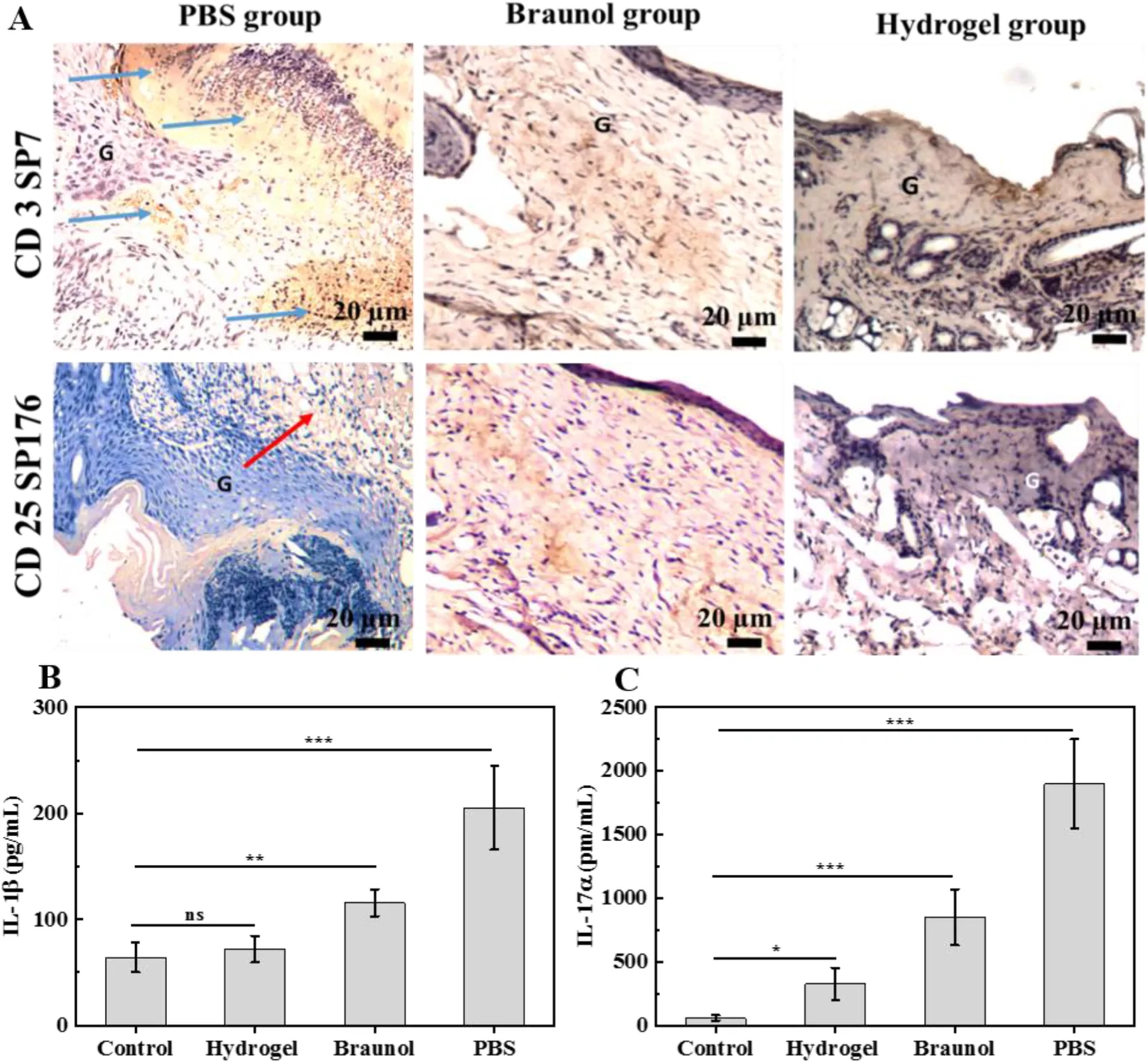
Evaluation of local immune cell infiltration and systemic inflammatory responses in a mouse *S. aureus*-infected full-thickness wound model. (A) Immunohistochemical localisation of CD3 and CD25 in wound tissues on day 10. Blue arrows indicate CD3-positive cells and the red arrow indicates CD25-positive cells near the granulation tissue (G). Quantification of serum IL-1β (B) and IL-17α (C) concentrations in healthy control mice and mice with *S. aureus*-infected wounds treated with PBS, Braunol, or hydrogel.

IL-1β is a key pro-inflammatory cytokine that plays a central role in innate immunity by regulating inflammatory responses to infection and tissue injury. During the early stages of wound healing, IL-1β increases vascular permeability and promotes leukocyte adhesion and recruitment, followed by activation of macrophages, neutrophils, lymphocytes, and other immune cells. Although these processes are essential for normal host defence, sustained IL-1β production contributes to chronic inflammation, tissue damage, and impaired wound healing. IL-1β also stimulates the production of downstream inflammatory mediators, including IL-17α, thereby amplifying the inflammatory response. IL-17α is a pro-inflammatory cytokine that serves as a key link between innate and adaptive immunity. It promotes the recruitment and activation of neutrophils at sites of infection and tissue injury, particularly at epithelial and mucosal barriers, and enhances the production of additional inflammatory mediators, including IL-6 and tumour necrosis factor-α (TNF-α). Persistent elevation of IL-17α has been associated with chronic inflammation and delayed wound healing, making both IL-1β and IL-17α relevant biomarkers for evaluating the inflammatory status of infected wounds.

To assess the systemic inflammatory response, serum concentrations of IL-1β and IL-17α were measured in mice treated with the hydrogel, Braunol, or PBS and compared with those of healthy mice from the same breeder used as non-infected controls. As shown in Fig. 11B, IL-1β concentrations in the hydrogel-treated (72 pg/mL) and Braunol-treated (115 pg/mL) groups remained close to those of healthy controls (65 pg/mL). In contrast, mice treated with PBS exhibited a markedly elevated IL-1β level (205 pg/mL), indicating a substantially stronger systemic inflammatory response.

IL-17α proved to be a more sensitive marker of infection-induced inflammation, with serum concentrations exceeding those of healthy controls in all experimental groups (Fig. 11C). Nevertheless, the hydrogel-treated mice exhibited considerably lower IL-17α levels (330 pg/mL) than the Braunol (850 pg/mL) and PBS (1900 pg/mL) groups, although values remained higher than those of healthy animals (60 pg/mL). Because IL-17α plays an important role in host defence against bacterial infection by promoting neutrophil recruitment and activation, its moderate elevation in the hydrogel group likely reflects an ongoing but controlled immune response, whereas the markedly higher levels observed in the PBS group are consistent with persistent infection and unresolved inflammation. This interpretation is supported by the pronounced neutrophilic infiltration observed histologically in the PBS-treated wounds.

Haematological analysis revealed leukocytosis, a characteristic feature of the inflammatory phase of wound healing, which was most pronounced in the PBS-treated group owing to the persistent *S. aureus* infection. This response was primarily reflected by increased total leukocyte (WBC), granulocyte (Gran), and lymphocyte (Lymph) counts. Although isolated animals in the Braunol and hydrogel groups exhibited mildly elevated haematological parameters, none showed clinical signs of systemic infection, and all maintained a good general health status. A modest increase in monocyte (Mon) counts was also observed, consistent with their well-established role in tissue repair, angiogenesis, and the transition from inflammation to regeneration. In contrast, red blood cell (RBC), haemoglobin (HGB), and platelet (PLT) counts remained within the normal reference ranges in all experimental groups (Table S1).

Microbiological analysis showed that only PBS-treated wounds exhibited polymicrobial contamination, including moderate growth of *E. coli* (25–100,000 CFU/mL), scant growth of *S. aureus* (<25,000 CFU/mL), and the presence of *Lactococcus lactis* (<25,000 CFU/mL), which is not considered a pathogenic species. In contrast, wound samples from mice treated with either the hydrogel or Braunol were sterile, indicating effective control of bacterial colonisation. Antimicrobial susceptibility testing indicated that the isolated strains were susceptible to linezolid, penicillin, ampicillin, amoxicillin–clavulanic acid, and vancomycin. These microbiological findings are consistent with the histopathological and immunological results, which demonstrated a markedly stronger inflammatory response in the PBS-treated group. The polymicrobial contamination of untreated wounds likely contributed to the persistent local inflammation and delayed healing observed in this group. In contrast, the hydrogel effectively prevented bacterial colonisation and supported wound healing with an efficacy comparable to that of the commercial antiseptic Braunol.

## 4 Conclusions

In this study, we developed and validated an injectable nano-enabled hydrogel dressing for chronic wound management. DOX/TA-functionalised lignin NPs (PLD NPs) were synthesised through a laccase-mediated sono-enzymatic process. Within the NPs formulation, DOX was incorporated at sub-inhibitory concentrations to selectively inhibit MMP activity without contributing to antibacterial effects, whereas the TA-functionalised polyphenolic shell provided the primary antioxidant, MPO-inhibitory, and antibacterial functions. Incorporation of the PLD NPs into a TCS matrix not only enabled rapid *in situ* self-assembly through dynamic supramolecular interactions but also enhanced antibacterial activity and inhibition of deleterious wound-associated enzymes. The resulting hydrogels exhibited stimuli-responsive NPs release under simulated chronic wound microenvironmental conditions (pH, H_2_O_2_, and lysozyme). Functionally, the hydrogels effectively inhibited MPO and MMP activity, scavenged ROS, suppressed bacterial growth and biofilm formation, and promoted fibroblast migration. The hydrogels also exhibited excellent cytocompatibility and protected fibroblasts from oxidative stress. In a full-thickness *S. aureus*-infected murine wound model, the TCS1/PLD(20) formulation demonstrated superior therapeutic efficacy compared with both PBS and Braunol. Histopathological, immunohistochemical, microbiological, haematological, and cytokine analyses consistently showed more effective infection control, accelerated tissue regeneration, reduced inflammatory responses, and advanced tissue remodelling characterised by hair follicle and skin appendage regeneration. The downregulation of IL-1β and IL-17α, together with reduced leukocytosis and complete bacterial eradication, further confirmed effective local immune modulation without evidence of systemic toxicity. Collectively, these findings demonstrate that the TCS/PLD hydrogel integrates infection control, modulation of the chronic wound microenvironment, and stimulation of tissue regeneration within a single multifunctional platform. This strategy represents a promising therapeutic approach for the treatment of chronic wounds and highlights the potential of sono-enzymatically engineered lignin NPs as versatile bioactive building blocks for next-generation regenerative biomaterials.

## CRediT authorship contribution statement

**Reza Ahmadi:** Writing – review & editing, Writing – original draft, Visualization, Validation, Software, Methodology, Investigation, Formal analysis, Data curation, Conceptualization. **Eva Ramon:** Writing – review & editing, Supervision, Project administration, Methodology, Investigation, Formal analysis. **Katerina Todorova:** Validation, Methodology, Investigation, Data curation. **Zdravka Petrova:** Validation, Methodology, Data curation. **Dolores Hinojosa-Caballero:** Validation, Methodology, Data curation. **Tzanko Tzanov:** Writing – review & editing, Writing – original draft, Supervision, Project administration, Funding acquisition, Formal analysis, Data curation, Conceptualization.

## Declaration of competing interest

The authors declare the following financial interests/personal relationships which may be considered as potential competing interests: Tzanko Tzanov reports financial support was provided by Spanish Ministry of Economy and Competitiveness (MINECO). If there are other authors, they declare that they have no known competing financial interests or personal relationships that could have appeared to influence the work reported in this paper.

## Data Availability

Data will be made available on request.
