# Supplementary material for: Sono-enzymatically engineered tannic acid/doxycycline-lignin nanoparticles enable self-assembled bioactive chitosan hydrogels for chronic wound healing
Source: Mater Today Bio. 2026 Oct 28;41:103792. doi: 10.1016/j.mtbio.2026.103792 (PMC13733470; doi:10.1016/j.mtbio.2026.103792)
Supplement: Supplementary file 1 [file mmc1.docx]

**Supporting Information**

**Sono-enzymatically engineered tannic acid/doxycycline-functionalised lignin nanoparticles drive self-assembly of bioactive chitosan hydrogels for chronic wound management**

Reza Ahmadi ^a^, Eva Ramon ^a^, Katerina Todorova ^b^, Zdravka Petrova ^b^, Dolores Hinojosa-Caballero ^c^ Tzanko Tzanov ^a,*^

^a^ Group of Molecular and Industrial Biotechnology, Department of Chemical Engineering, Universitat Politècnica de Catalunya, Rambla Sant Nebridi, 22, Terrassa 08222, Spain

^b^ Institute of Experimental Morphology, Pathology and Anthropology with Museum, Bulgarian Academy of Sciences, Sofia, Bulgaria, Acad. G. Bonchev Str., Bl. 25, 1113, Sofia, Bulgaria

^c^ Unitat de Ferides Complexes, Consorci Sanitari de Terrassa Hospital de Terrassa, Ctra. Torrebonica, s/n, 08227 Terrassa, Spain

^*^ Corresponding authors

Email: [tzanko.tzanov@upc.edu](mailto:tzanko.tzanov@upc.edu)

Figure S1. Characterisation of PLD NPs. (A) Hydrodynamic particle size distribution (average diameter of 310 nm). (B) Zeta potential distribution (−32.5 mV). (C) XPS spectra of PLD NPs showing the surface chemical composition, including the C 1s, O 1s, and N 1s regions. (D) FESEM micrograph of PLD NPs. (E) Intracellular ROS generation in *S. aureus* and *P. aeruginosa* induced by PLD NPs at various concentrations.

Figure S2. Raman spectroscopy analysis of the CS and TCS.

Figure S3. Rheological characterisation of self-assembled TCS/PLD hydrogels at 37 °C. (A and B) Three-interval thixotropy test (3iTT). (C and D) Frequency sweep analysis. Panels A and C correspond to hydrogel formulations containing varying concentrations of PLD NPs, whereas panels B and D correspond to formulations with varying degrees of thiolation. Oscillatory strain sweep measurements of TCS1/PLD(10) hydrogel following 7-day incubation at 37 °C under different pH conditions (pH 4–9) (E) and in PBS, wound exudate, H₂O₂, and lysozyme-containing media (F).

Figure S4. Physicochemical and protein-interaction studies of TCS/PLD hydrogels. (A) Zeta potential of hydrogel formulations measured across a pH range of 4–9. (B) Zn²⁺ chelation by polymer matrix as a function of thiol content (TCS1, TCS2, and TCS3). (C) Time-dependent BSA adsorption profiles by hydrogels during incubation from 1 to 72 h at 37 °C and pH 7. (D) FTIR spectra of BSA, pristine hydrogel, and hydrogel after 72 h incubation in BSA-containing buffer.

Figure S5. Fluorescence microscopy images of fibroblast migration in a scratch wound assay. Representative images of cell migration into the scratch area at (A) 0 h, (B) 24 h, and (C) 48 h. For each time point, images were obtained from three different regions of the scratch (n = 3).

Figure S6. Mouse wound treated with PBS only stained with H&E. Increased numbers of white blood cells and formation of lymphocyte-neutrophile demarcation line /white arrows/ under the granulation tissue, infiltrated with inflammatory cells too. Yellow arrow – deep dermal inflammatory and oedema process.

| **Parameters** | **Ref. Range** | **Hydrogel group**  **Mouse 1, 2, 3, 4** | **Braunol group**  **Mouse 1, 2, 3, 4** | **PBS group**  **Mouse 1, 2, 3, 4** |
| --- | --- | --- | --- | --- |
| WBC 10^9^/L | 0.8 - 6.8 | 6.8; **H** 6.9; 6.2; 6.3 | 6.7; 5.5; 6.7; 6.2 | **H** 10.5 ; **H** 11.2; **H** 8.3; **H** 8.5 |
| Lymph. 10^9^/L | 0.7 - 5.7 | 4.7; 5.1; 5.2; 5.1 | 5.4; 4.2; 5.1; 5.6 | **H** 6.6; **H** 7.1; 5.7; 5.7 |
| Mon. 10^9^/L | 0.0 – 0.3 | **H** 0.4; 0.3; **H** 0.4; 0.3 | 0.3; **H** 0.4; **H** 0.5; 0.2 | **H** 1.4; 0.3; **H** 0.4; **H** 0.5 |
| Gran. 10^9^/L | 0.1 – 1.8 | 1.7; 1.5; 0.6; 0.9 | 1.0; 0.9; 1.1; 0.4 | **H** 2.5; 3.8; **H** 2.2; **H** 2.3 |
| RBC 10^12^/L | 6.36 – 9.42 | 8.1; 7.9; 9.1; 8.5 | 8.83; 8.5; 7.96; 8.9 | 7.9; 8.3; 7.9; 8.0 |
| HGB g/L | 110 – 143 | 129; 124; 134; 121 | 126; 157; 133; 139 | 120; 154; 118; 135 |
| PLT 10^9^/L | 450 – 1590 | 879; 960; 839; 952 | 1003; 948; 862; 1050 | 850; 1044; 892; 969 |

Table S1. Hematological analyses of blood parameters of mice from all groups.

* H indicates a value exceeding the corresponding reference range.
